# Design Rules for Binary Bisamide Gelators: toward Gels with Tailor-Made Structures and Properties

**DOI:** 10.1021/acs.langmuir.3c01487

**Published:** 2023-08-14

**Authors:** Elmira Ghanbari, Stephen J. Picken, Jan H. van Esch

**Affiliations:** Advanced Soft Matter (ASM) Group, Chemical Engineering Department, Faculty of Applied Science (TNW), Delft University of Technology, 2629 HZ, Delft, The Netherlands

## Abstract

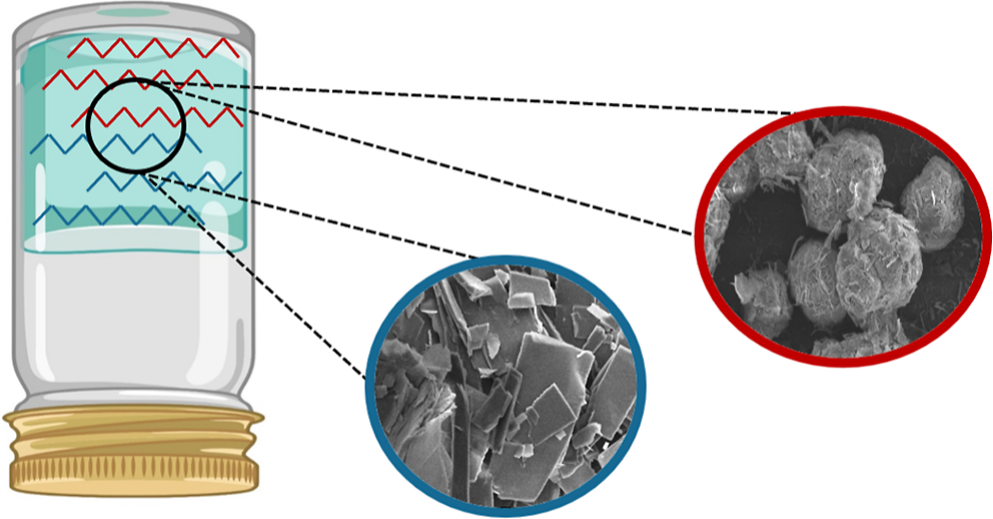

This study intends to develop design rules for binary mixture of gelators that govern their assembly behavior and subsequently explore the impact of their supramolecular assembly patterns on the gels’ rheological properties. To achieve these goals, *n*BA gelators with odd and even parities [*n*-methylene spacers between the amide groups (*n* = 5–10) and 17 carbons at each end] were blended at different ratios. Such bisamides with simple structures were selected to study because their different spacer lengths offer the possibility to have matching or non-matching hydrogen bonds. The results show that the assembly behavior of binary mixtures of bisamide gelators is the same in the solid and gel states. Binary mixtures of gelators, which only differ two methylene moieties in the spacer length, form compounds and co-assemble into fibers and sheets observed for (5BA)_1_(7BA)_1_ and (6BA)_1_(8BA)_1_ mixtures, respectively. Binary gelator mixtures of the same parity and a larger spacer length difference still lead to mixing for the odd parity couple (5BA)_1_(9BA)_1)_, but to partial phase separation for the even parity mixture (6BA)_1_(10BA)_1_. Binary mixtures of gelators of different parities gave complete phase separation in the solid state, and self-sorted gels consisting of discrete fibers and sheets in the gels of (5BA)_3_(6BA)_1_ and (5BA)_3_(10BA)_1_. The even–even binary gels (20 wt %) consisting of co-assembled sheets show higher *G*′ than odd–odd binary gels (20 wt %) consisting of co-assembled fibers. In general, the self-sorting of odd and even molecules into the separate primary structures results in a dramatic decrease of *G*′ compared to the co-assembled gels (20 wt %), except for (5BA)_1_(9BA)_1_ gel (20 wt %). It might be due to larger woven spheres in (5BA)_1_(9BA)_1_ gel (20 wt %), which probably have a less entangled gel network.

## 1 Introduction

Supramolecular gels are composed of two main components, a small molecule like low-molecular-weight gelators (LMWGs) and a solvent system. During the gelation process, gelator molecules respond to external stimuli, such as temperatures. Many LMWGs depending on their chemical structures are thermally triggered,^1−6^ the LMWGs are dissolved in a solvent upon heating to higher temperatures and usually self-assemble into primary structures by cooling the solution to below the gelation transition temperature.

In the course of cooling when sufficient undercooling has been reached, the gelator molecules assemble through non-covalent bonds to build primary structures, such as tapes, rods, fibers, sheets, and cylinders.^7^ The properties of these gels are governed by both the primary structures and their interactions in a mesoscopic scale.^8,9^ In fact, these primary structures are able to efficiently immobilize the solvent if they form a three-dimensional entangled network.^9−11^

Initially, structurally diverse LMWGs were developed based on saccharides,^12^ peptides, ureas, amides,^13−15^ nucleobases,^16^ steroid derivatives,^17^ dendrimers,^18^ etc. to produce supramolecular gels for many potential applications.^19−29^ In recent years, the field of single LMWGs was expanded from ab initio synthesis of new gelators with new functionalities toward designing multicomponent systems to introduce more flexibility in the gel system for different purposes, generally aiming at controlling the assembly pattern and study its effect on the gel properties.^30^ For instance, for biomedical applications, the main LMWGs in the system are used to develop the supporting network structure, while a second assembly moiety is added to improve the cell adhesion to the network matrix.^31^

Primary structures in multicomponent gels can form either via the co-assembly of gelators with alternating order or random organizations or via their self-sorting into discrete structures.^32−34^ It is also likely that the final gel structure is the mixture of all these assembly modes.^35^ Another advantage of using multicomponent gelator systems is the reinforcement in the mechanical properties of the final gel formulation by inducing co-assembly of the gelator molecules^36^ or via encouraging the self-sorting of gelator molecules in pH-triggered hydrogels where each gelator component responds to a certain pH.^37^

Here, the aim is to develop design rules for the binary mixture of gelators that govern the mixing or phase separation behavior and, subsequently, explore its impact on the phase behavior and their relation to the gel properties. For that purpose, the binary mixtures of bisamide gelators with odd–even spacer lengths were used. These gelators were chemically designed to have the simplest structure, *n*-methylene spacers between the amide groups (*n* = 5–10) and 17 carbons at each end, which makes them ideal model systems to tune mixing or phase separation behavior via hydrogen bond complementarity. Our hypothesis is that the systematic addition of a *n*BA gelator with different parities or spacer lengths to the parent *n*BA gelator, partial, or matching complementary interactions between the gelator molecules can determine their phase behavior.

To achieve the above-mentioned objectives, these systems were studied in multiple steps. Previously, the molecular arrangement and hydrogen bonding pattern of a series of single bisamide gelators with different spacer lengths have been fully investigated,^38^ and also their gelation behavior and the odd–even effect on their rheological properties have been addressed.^39^ In this work, binary mixtures of these bisamide gelators in the solid state and the gels were studied. The effects of differences in the spacer length and parity on the solid-state phase behavior and the gel structure and thermal properties were studied. Finally, the rheological properties of the gels from the binary gelator mixtures have been studied in relation to the phase behavior as well as the rheological properties of the gels from the single bisamides.

## 2 Materials and Methods

### 2.1 Binary Mixtures of BA Gelators

**Figure 1:**
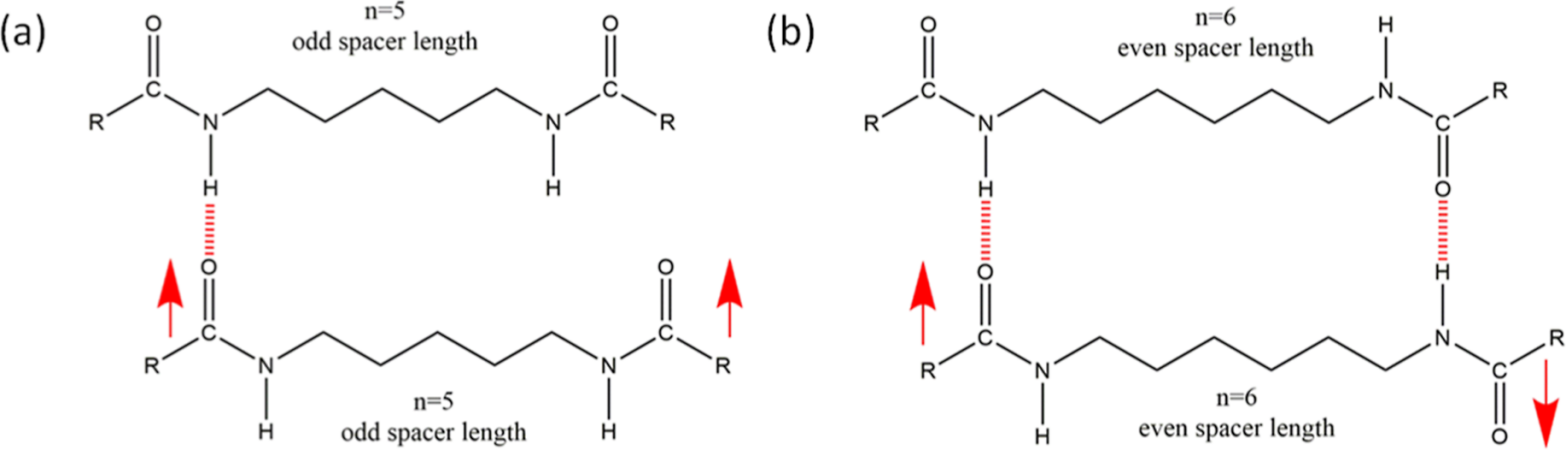
Chemical structure of single bisamide gelators (*n*BAs) with the (CH_2_)_*n*_ spacer between the amide groups: (a) 5BA with a parallel arrangement and (b) 6BA with an anti-parallel arrangement.

The synthesis and characterization of single *n*BA gelators, with *n*-methylene spacers between the amide groups (*n* varies from 5 to 10) and 17 carbons at each end, were described in our previous research.^38^ The single *n*BA gelators were notated according to the number of carbon atoms in the spacer between the amide groups (*n*) with “BA” as the suffix for “bisamide gelator”. The general chemical structure of *n*BA gelators is shown in Figure 1 for 5BA and 6BA as examples of odd and even *n*BA gelators, respectively.^38^

The binary bisamides were prepared by systematic blending (odd–odd, even–even, and odd–even spacer length) of two single *n*BA gelators (Table 1). The single gelators were heated to their melting points and were stirred isothermally just above their melting points to ensure that the gelators are uniformly mixed. After mixing for 15 min, the mixtures were cooled down to room temperature. Binary gelators were prepared at five different mole ratios (1:7, 1:3, 1:1, 3:1, and 7:1). The binary gelators were named by taking the notation of their single nBA gelators with their assigned ratios as the subscript; for example, the blend of 5BA and 6BA in the 1:7 ratio is notated as (5BA)_1_(6BA)_7_.

**Table 1:** Systematically Blended Bisamide Gelators Based on the Parity of Spacer Length to Produce Binary BAs at five Different Ratios

blending based on parity	directionality of amide groups	single components	difference in spacer length	molar ratios
odd–odd	parallel–parallel	5BA and 7BA	2	
		5BA and 9BA	4	1:7
even–even	antiparallel–antiparallel	6BA and 8BA	2	1:3
		6BA and 10BA	4	1:1
odd–even	parallel–antiparallel	5BA and 6BA	1	3:1
		5BA and 10BA	5	7:1

### 2.2 Gel Preparation

The gelation behavior of single *n*BA gelators was investigated in our former research.^39^ To prepare the gels from binary BA gelators, binary gelators were first ground into powders. Then, they were weighed (20 wt %) and dispersed in xylene as the solvent. The gelator–solvent mixtures were heated to 120 °C using a heating block while stirring using magnetic bars at 500 rpm. Once the mixtures became transparent, the vials were taken out and cooled down to the ambient temperature. A tube inversion test^40^ was conducted as a quick assessment of gel formation after cooling down and after 72 h.

### 2.3 Differential Scanning Calorimetry

The melting transition of gelators and gel–sol transition temperatures of gel samples were measured using a Perkin Elmer-Pyris Diamond differential scanning calorimeter with two 1(g)-furnaces (working on the power-compensation temperature null principle with a temperature accuracy/precision of ±0.1 °C/± 0.01 °C and calorimetry accuracy/precision of <± 1%/<± 0.1%). Nitrogen (99.99% purity) was used to purge the interior system at a rate of 50 (mL min^–1^). Temperature and heat flow calibration were done before each measurement under the same condition as the measurement of the samples.

For the solid binary gelator mixtures, about 6 ± 1 mg of binary bisamide gelator was placed in a 40 μL aluminium sample pan with an ambient maximum pressure. The sample pan and the identical reference pan were both covered by aluminium lids. The sealed pans were placed in the furnaces of the DSC apparatus. Both pans were heated from room temperature to at least 30 °C above the temperature range of interest. Isothermal melting was followed by a fixed cooling cycle preceding a second heating cycle, all scans at the rate of 10 K min^–1^. A similar procedure was followed for the gel samples: a gel sample (8 ± 1 mg) in a 40 μL stainless-steel sample pan and an identical empty pan as reference, both covered by stainless steel lids, were heated at 5 K min^–1^ to 130 °C. The first heating cycle to eliminate the sample thermal history was followed by a cooling cycle 5 K min^–1^ to the ambient temperature. Finally, a second heating cycle was run at 5 K min^–1^ to 130 °C to study the thermal properties of the samples. To assure the thermal equilibrium of the sample, the samples were kept isothermally for 2 min at the end of each temperature scan.

### 2.4 DSC_N_(*T*) Analytical Model and Curve Fitting

DSC_N_(*T*) function has been developed in our research group^41^ and was extended for binary gelators, which show two endothermic peaks in their second heating DSC traces (eq 1).
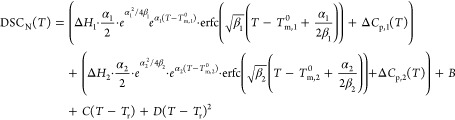
1
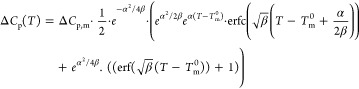
2

DSC_N_(*T*) is based on an assumed Arrhenius function taking the crystal size distribution into account together with instrumental and sample-related peak broadening. Relying on DSC_N_(*T*), melting point (*T*_m_^0^) and enthalpy of fusion (Δ*H*) of each phase in binary gelators can be obtained much more accurately. In eq 1, Δ*H*_1_ and Δ*H*_2_ are the coefficients representing the change in enthalpy associated with the phase transition of each peak, *T*_m,1_^0^ and *T*_m,2_^0^ are the equilibrium temperatures of the phase transitions. The strength of the linearized Arrhenius function (α) is related to the crystal size distribution. Parameter β describes the Gaussian peak broadening distribution of the peak due to the peak broadening, which is related to the peak width in the declining edge. The difference between the heat capacity of the pre- and post-transition states is represented by Δ*C*_p,m_. The parameters *B*, *C*, and *D* correct for the baseline features; the offset, linear slope, and second-order curvature, respectively. This model captures the shape of experimental DSC peaks of binary bisamides, where *T*_r_ is a reference temperature for the overall curved baseline. The non-linear curve fitting of DSC_N_(*T*) to the experimental DSC traces of binary compounds was done using the Python 3 programming language, which has been described comprehensively in our previous work.^41^ To assure the accuracy of thermal properties obtained from DSC measurements, at least three samples with similar weights were measured under the same condition and the data were analyzed after normalization per weight of the sample. The standard deviation of the thermal properties, melting temperature, enthalpy of fusion, and heat capacity change, and the other fit parameters were obtained by fitting the analytical model DSC_N_(*T*) to the three sets of raw data, which contains the experimental error along with the fitting procedure error. The fitting deviation for each parameter was obtained from the residuals of non-linear least squares (NLLS). The reported error margins of the fit parameters in Tables S1–S12 are the residuals of NLLS rounded to two digits.

### 2.5 X-ray Diffraction

To compare the crystal structures of single gelators and their gels with binary BA systems, X-ray diffraction (XRD) patterns of these gels were studied. A dab of sample was placed onto the standard PMMA sample holders. The measurement on binary systems was conducted under the same condition as single *n*BA gelators and gels:^38,39^ a Bruker D8 ADVANCE ECO diffractometer in the Bragg–Brentano geometry equipped with a Cu X-ray source (K_α1_ = 1.54060 Å and K_α2_ = 1.54439 Å) and LYNXEYE-XE-T position-sensitive detector was operated at room temperature. A knife-edge was embedded to reduce the background due to the scattering of the primary beam. The diffraction patterns were recorded from 0.6 to 50° (2θ) with a step size of 0.01° and measuring time of 0.5 s per step.

### 2.6 Scanning Electron Microscopy

Samples for scanning electron microscopy (SEM) were prepared by placing a small amount of freshly prepared gels on an aluminum foil covering a microscope glass. To get rid of the solvent, the samples were dried in a VT6025 vacuum oven (Thermo Electron Corporation) for 3 h at 60 °C. Due to the low volatility of xylene, applying this temperature in addition to the vacuum drying is essential. The temperature 60 °C has been chosen safely below the melting point of the gel sample and its constituents to avoid any morphological change. To improve conductivity for better image quality, the samples were placed in a benchtop SEM sputter coater to be coated with a thin layer of gold particles at 20 mA for 30 s. The morphology of the binary gels was observed by using a JEOL JSM 6010LA scanning electron microscope with an accelerating voltage of 8 kV. The images were recorded at different magnifications (500×, 1000×, and 2500×) in the secondary electron image mode, WD10 mm, and SS40.

### 2.7 Rheology

Rheological measurements were carried out on a DISCOVERY HR-3 hybrid rheometer (TA Instrument). Parallel steel plates (with diameter of 40 mm) and Peltier plate steel-999580 were used. Geometry gap between the plates was set at 500 μm. Zero gap was determined after calibration of inertia, friction, and rotational mapping. A freshly prepared gel sample (1.0 ± 0.2 g) was placed evenly on the bottom plate, and then the parallel plate was lowered to the set geometry gap. All the measurements were performed at 25 °C. To avoid solvent evaporation during the measurement, a few drops of xylene were added on a solvent trap covering the gap prior to each measurement. Before frequency sweep, an amplitude sweep test from 0.001 to 1 s^–1^ strain rate at 1 Hz was conducted on each sample to find the linear viscoelastic region. The strain rate was set to 0.01 s^–1^ selected from the linear viscoelastic region. Frequency sweep measurement was carried out setting angular frequency from 0.1 to 100 rad·s^–1^ and the strain 0.01 was applied to measure the storage modulus (*G*′) and loss modulus (*G*″). To compare the modulus of binary and single *n*BA gels, the values of *G*′ at a constant frequency (ω = 10 rad·s^–1^) for all gels were chosen. The final *G*′ values are the average of the *G*′ from the measurement of three independent samples. To compare the rheological properties of the single and binary gels, the binary gel samples were prepared and measured under the same conditions as the single *n*BA gels.^39^

## 3 Results and Discussion

Here, in Section 3.1, the molecular arrangement of binary bisamide gelators in the solid will be discussed and subsequently the supramolecular assembly in the gel state will be explained in Section 3.2. Finally, the effect of complementarity of hydrogen bonding on the rheological properties will be described in Section 3.3.

### 3.1 Binary Gelators in the Solid State

To investigate the effect of the addition of the second *n*BA gelator on the thermal properties of the parent gelator, melting transitions of single gelators and their binary compositions were studied by DSC. The experimental DSC traces were fitted using the DSC_N_(*T*) function for single^38^ and binary systems (eq 1) to obtain the melting point (*T*_m_^0^) and enthalpy of fusion (Δ*H*) for each phase accurately. The fit parameters for all gelators and their binary compounds are listed in the tables in the Supporting Information file.

#### 3.1.1 Odd–Odd Binary Gelators

**Figure 2:**
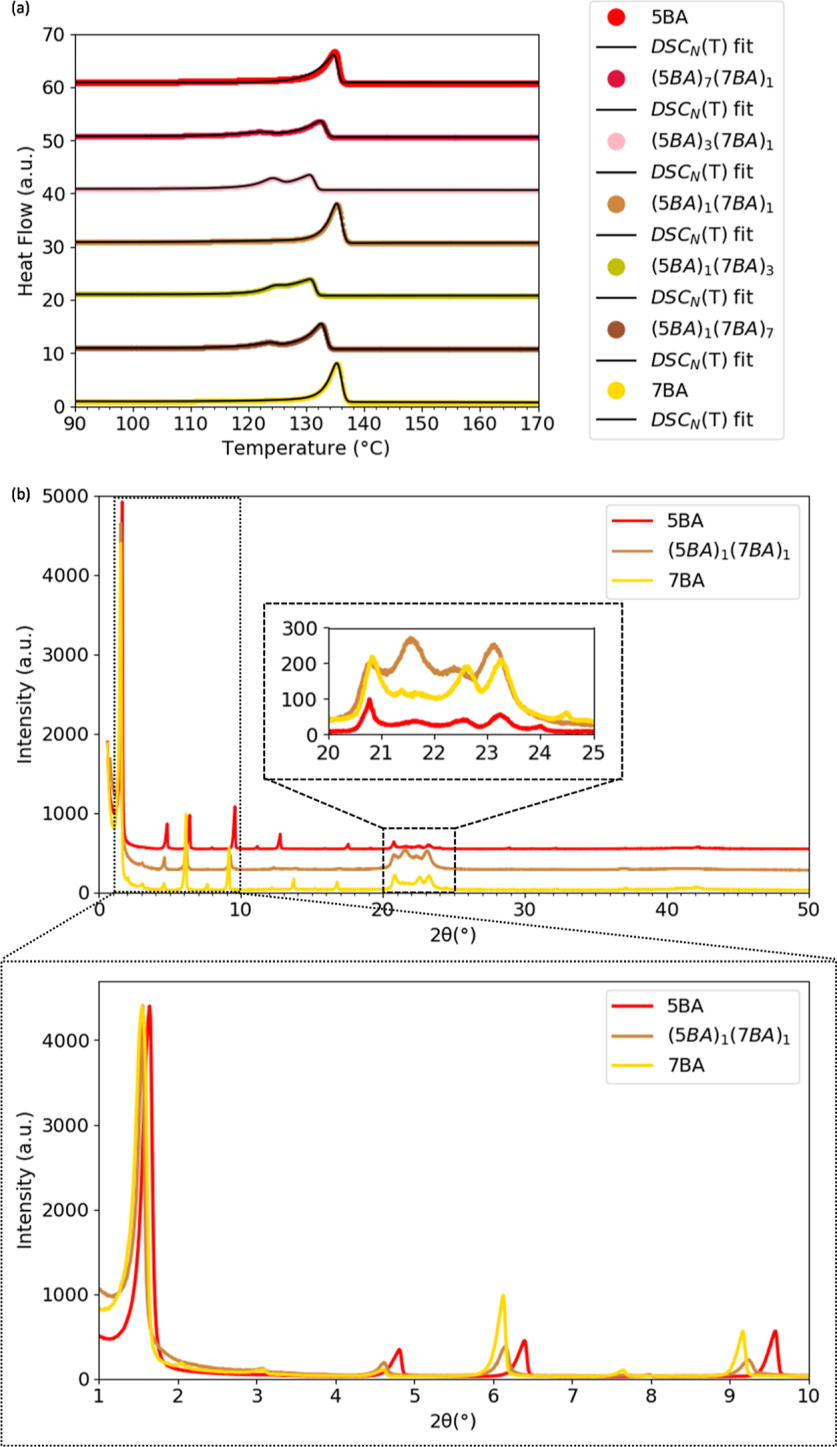
Phase behavior of 5BA7BA gelators: (a) DSC_N_(*T*) fit to the second heating DSC traces of 5BA7BA at different mixing ratios (the traces and fits were shifted vertically for clarity) and (b) XRD patterns of (5BA)_1_(7BA)_1_ mixtures in comparison to single 5BA and 7BA gelators (curves were normalized to the highest intensity), the insets magnify high-angle (20–25° (2θ)) and low-angle (0–10°(2θ)) regions.

The DSC traces and DSC_N_(*T*) fits of 5BA, 7BA, and their binary compositions at different ratios are shown in Figure 2a. At all non-equimolar binary compositions, two separate peaks are observed while the equimolar mixture (5BA)_1_(7BA)_1_ shows a single peak. The *T*_m_^0^ of both phases in binary 5BA7BA gelators are at lower temperatures (Table S1) compared to the single gelators 5BA and 7BA gelators, with *T*_m_^0^ of 135.5 and 136.1 °C, respectively. However, *T*_m_^0^ of (5BA)_1_(7BA)_1_ has increased to 136.1 °C, close to the *T*_m_^0^ of single 5BA and 7BA. The seemingly single melting transition of (5BA)_1_(7BA)_1_ could be due to either two single overlapping peaks or the formation of a new compound. To find out, the XRD pattern of (5BA)_1_(7BA)_1_ was compared to the diffraction patterns of the corresponding single compounds, 5BA and 7BA, as previously studied.^38^Figure 2b shows that the first-order reflection of (5BA)_1_(7BA)_1_ is a single reflection at 1.55° (2θ) at different 2θ positions from the 001 reflection of 5BA, at 1.63° (2θ), but at the same position as the 001 reflection of 7BA, at 1.55° (2θ). At higher angles [20–25° (2θ)], the reflections of (5BA)_1_(7BA)_1_ do not correspond to the reflections of single 5BA and single 7BA and have shifted toward lower angles. This indicates the formation of a single phase with a supramolecular arrangement very similar to the crystal structure of 7BA.

Increasing the difference between the spacer length of binary odd–odd gelators from 2 (in 5BA7BA) to 4 (in 5BA9BA) shows the similar trend of DSC traces as 5BA7BA compositions (Figure S1a): two separate melting peaks for a non-equimolar ratio while a single melting peak for (5BA)_1_(9BA)_1_. However, on the contrary to (5BA)_1_(7BA)_1_, the single melting transition of (5BA)_1_(9BA)_1_ has shifted to the lower temperature (126.5 °C) compared to its corresponding single gelators, 5BA (135.5 °C) and 9BA (132.50 °C). As shown in Figure S1b, the single 001 reflection of (5BA)_1_(9BA)_1_ is a broad peak at 1.55° (2θ) between the reflections of single 5BA [1.63° (2θ)] and 9BA [1.46° (2θ)] implying that they have formed the compound (5BA)_1_(9BA)_1_.^38^

#### 3.1.2 Even–Even Binary Gelators

For binary gelators of 6BA8BA, where 6BA and 8BA are two spacer lengths apart and have the same parity, the general trend of the DSC thermogram is very similar to 5BA7BA with the same above-mentioned characteristics (Figure S2a); at non-stoichiometric ratios, two peaks but they appear to merge into a single melting transition at 1:1 ratio. This single melting transition is observed for (6BA)_1_(8BA)_1_ at 143.5 °C, which is lower than the *T*_m_^0^ of the parent gelators, single 6BA (147.5 °C) and 8BA ( 143.5 °C) but higher than the *T*_m_^0^ of the mixtures. Similar to the XRD pattern of (5BA)_1_(7BA)_1_, where the first-order reflection occurs at the same position as the 001 reflection of 7BA, the XRD pattern of (6BA)_1_(8BA)_1_ in Figure S2b shows a single reflection at 2.00° (2θ), which is at the exact same position as the 001 reflection of 8BA. Similar *T*_m_^0^ and XRD 001 reflection of (6BA)_1_(8BA)_1_ to the those of single 8BA indicates that a compound has formed at the 1:1 ratio, which has a similar crystal structure to 8BA.

**Figure 3:**
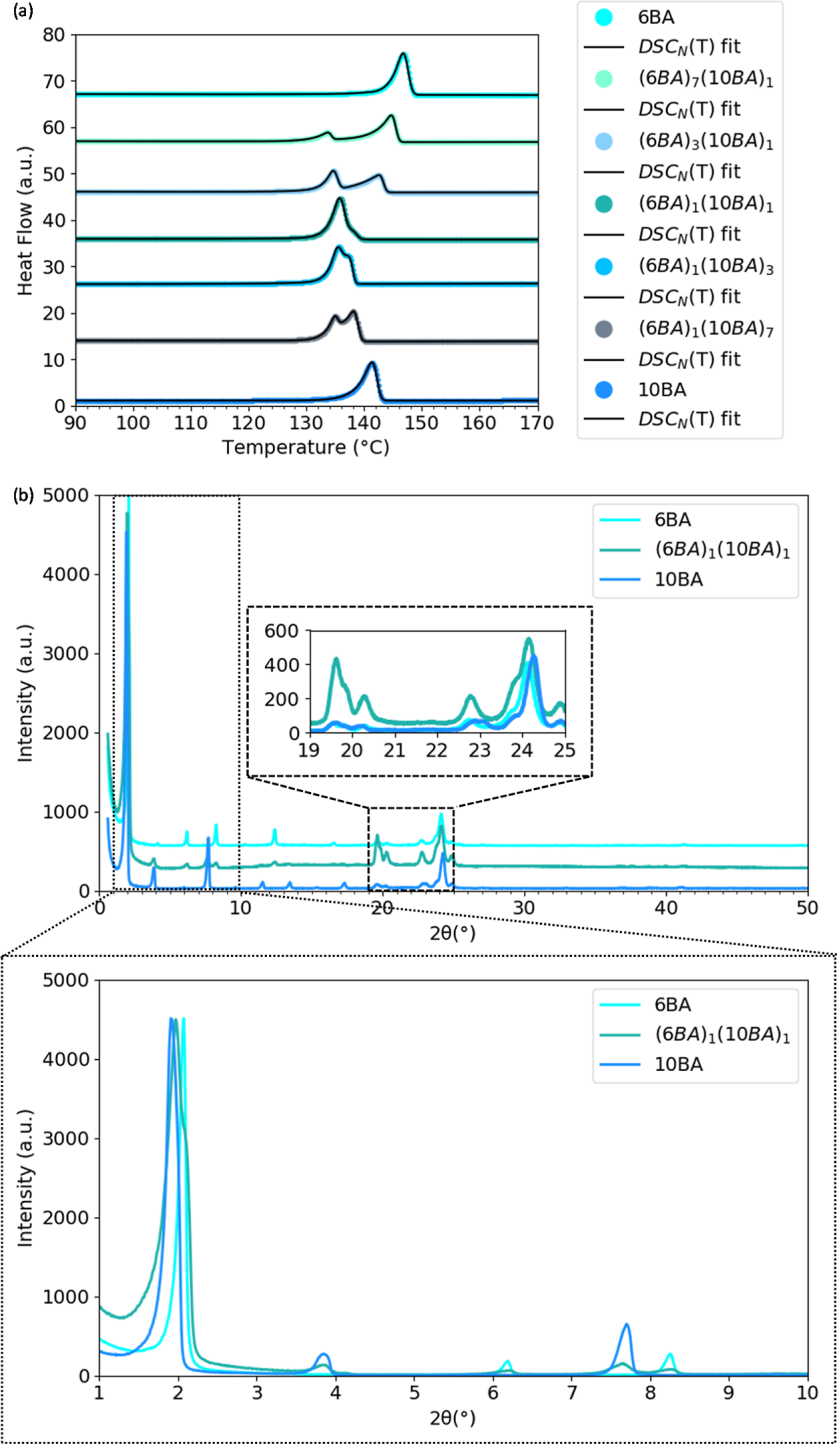
Phase behavior of 6BA10BA gelators: (a) Second heating traces for various mixing ratios 6BA and 10BA and DSC_N_(*T*) fits on the experimental traces (the traces and fits were shifted vertically for clarity) and (b) XRD patterns of (5BA)_1_(6BA)_1_ in comparison to single 6BA and 10BA gelators (curves were normalized to the highest intensity), the insets magnify high-angle [20–25° (2θ)] and low-angle [0–10° (2θ)] regions.

Keeping the parity the same and increasing the difference in the spacer length to 6BA10BA, all compositions of 6BA10BA, including the equimolar composition show two melting peaks in the DSC thermogram (Figure 3a) implying that 6BA and 10BA have less tendency to mix compared to 6BA8BA. As shown in Figure 3b shows, the first two reflections of (6BA)_1_(10BA)_1_ at 1.97° and 2.08° (2θ) are comparable to the 001 reflections of single 6BA at 2.08° (2θ) and 10BA at 1.91° (2θ). The XRD pattern of (6BA)_1_(10BA)_1_ is approximately a linear superposition of the single 6BA and single 10BA XRD patterns, which confirms that these molecules have mostly phase separated. This can be due to the larger difference in the spacer length of 6BA and 10BA causing a mismatch of molecules for the paired hydrogen bonding observed for single even bisamide gelators.^38^

#### 3.1.3 Odd–Even Binary Gelators

**Figure 4:**
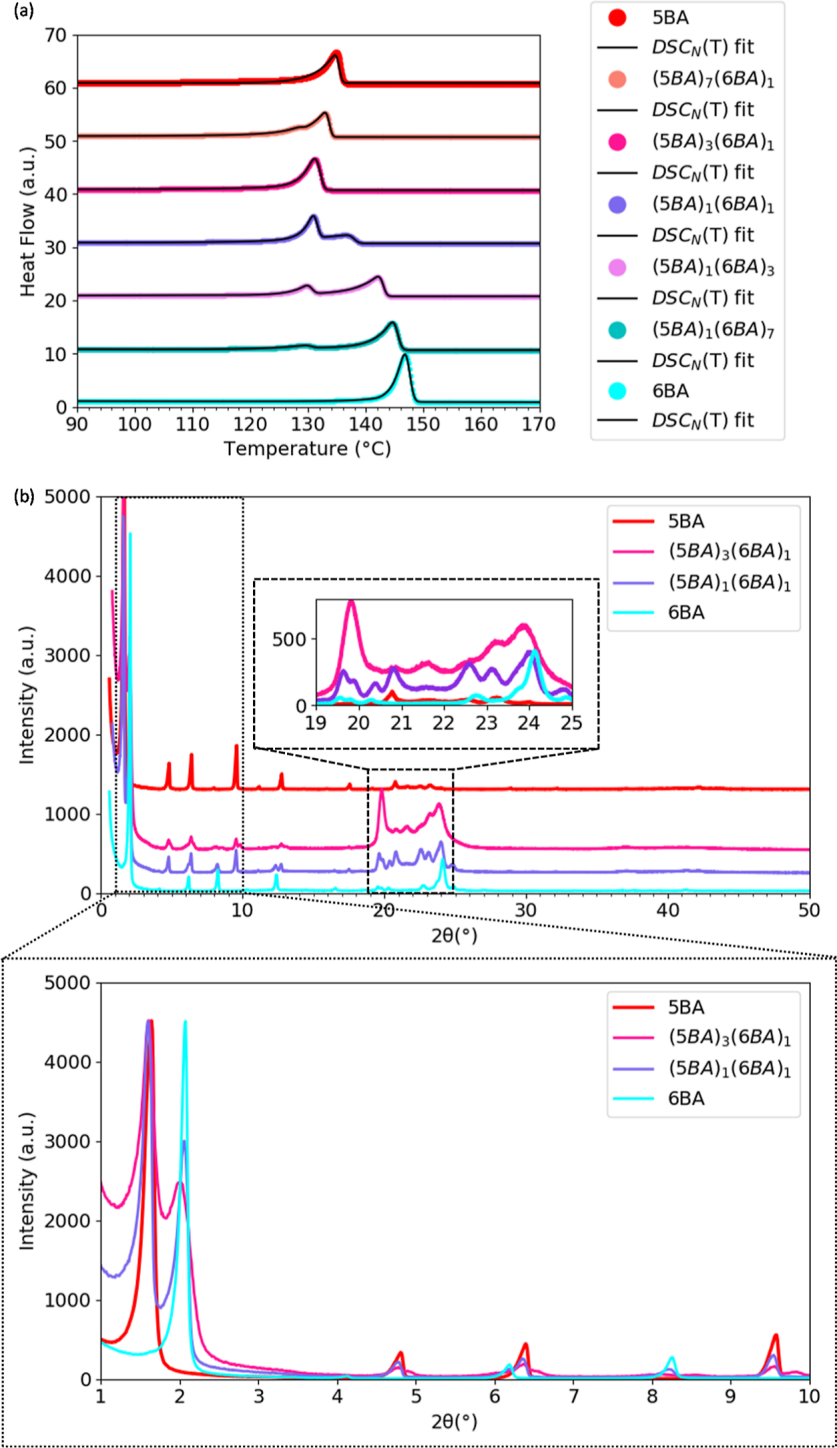
Phase behavior of 5BA6BA gelators: (a) second heating traces for various mixing ratios 5BA and 6BA and DSC_N_(*T*) fits on the experimental traces (the traces and fits were shifted vertically for clarity), (b) XRD patterns of (5BA)_3_(6BA)_1_ in comparison to single 5BA and 6BA gelators and binary (5BA)_1_(6BA)_1_, which has two distinct melting peaks and shows split first-order reflections (curves were normalized to the highest intensity), the insets magnify high-angle [20–25° (2θ)] and low-angle [0–10° (2θ)] regions.

The DSC thermogram of 5BA and 6BA mixtures at different compositions (Figure 4a) shows double melting transitions for all compositions except for (5BA)_3_(6BA)_1_. (5BA)_3_(6BA)_1_ melts at 131.0 °C, which is lower than single 5BA and 6BA (Table S5). The seemingly single melting transition of (5BA)_3_(6BA)_1_ at 136.0 °C could be due to either two single overlapping peaks or the formation of a new phase. To find out, the XRD pattern of (5BA)_3_(6BA)_1_ was compared with the patterns of 5BA, 6BA, and (5BA)_1_(6BA)_1_. Figure 4b shows the splitting of the first-order reflection both for (5BA)_3_(6BA)_1_, with single melting peak, and (5BA)_1_(6BA)_1_, with double peaks. The first and second reflections of (5BA)_3_(6BA)_1_ have shifted to lower positions of 1.61° (2θ) and 1.99° (2θ) with respect to the first-order reflections of 5BA [at 1.64° (2θ)] and single 6BA [at 2.07° (2θ)]. These two reflections are at the same angles as the two first reflections of (5BA)_1_(6BA)_1_. This demonstrates that the single melting transition is the result of two overlapping yet separated phases.

Although 5BA10BA has a larger difference in the spacer length than 5BA6BA, its DSC thermogram shows a similar trend to 5BA6BA (Figure S3a): all binary compositions show two distinct melting peaks. The presence of two completely distinct peak is less obvious for (5BA)_3_(10BA)_1_ due to two mostly overlapping peaks. The first reflection of (5BA)_3_(10BA)_1_ is at 1.64° (2θ), at the same position as the 001 reflection of single 5BA, and the second reflection has shifted to a lower angle [1.86° (2θ)] than the 001 reflection of 10BA at 1.90° (2θ). These two reflections are at the exact angles as the two reflections of (5BA)_1_(10BA)_1_, where two distinct melting peaks are observed in Figure S3a. Similar to (5BA)_3_(6BA)_1_, phase separation occurs for (5BA)_3_(10BA)_1_

### 3.2 Phase Diagram

**Figure 5:**
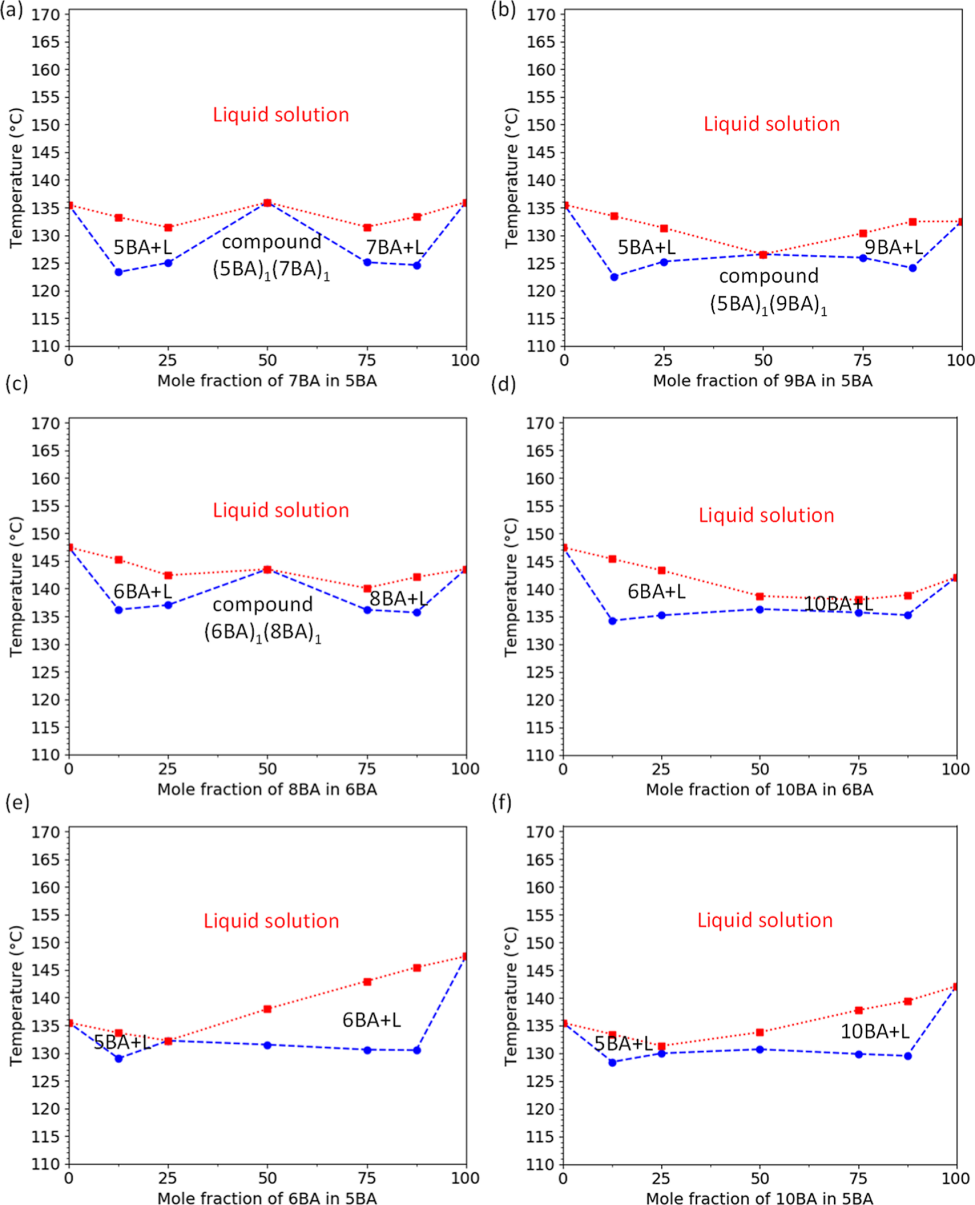
Temperature-composition phase diagrams of binary BAs constructed from *T*_m_^0^ obtained from the fitting of DSC_N_(*T*) to the experimental traces of single *n*BA gelators and their binary blends at different compositions (the blue and red curves are the *T*_m_^0^ of the first and second phases, respectively): (a) 5BA7BA, (b) 5BA9BA, (c) 6BA8BA, (d) 6BA10BA, (e) 5BA6BA, and (f) 5BA10BA showing compound formation for 5BA7BA and 6BA8BA and mixtures with lower *T*_m_^0^ for (5BA)_1_(9BA)_1_ and (6BA)_1_(10BA)_1_. Despite odd–odd and even–even binary gelators, the mixtures of 5BA6BA and 5BA10BA with lower *T*_m_^0^ compared to single gelators occur approximately at the 3:1 composition.

To gain more insights on how the addition of the second *n*BA affects the *T*_m_^0^ of the parent *n*BA, the phase diagrams of all *n*BA gelators (temperature vs composition) are constructed and shown in Figure 5a–f. The *T*_m_^0^ of all phases at different compositions were obtained from the fitting of DSC_N_(*T*) to their DSC experimental traces.

#### 3.2.1 Odd–Odd Binary Gelators

The phase diagram of 5BA7BA (Figure 5a) shows that 5BA and 7BA form a compound, (5BA)_1_(7BA)_1_ with nearly the same *T*_m_^0^ as the single gelators (Table S1). The compound formation of 5BA and 7BA with the same parity and close spacer length can be due to their similar crystal structure.^38^ A closer look into the phase diagram of 5BA7BA shows that (5BA)_1_(7BA)_1_ forms eutectic mixtures with single 5BA and single 7BA, respectively, at around 12.5 and 87.5% of 7BA in 5BA.

As shown in Figure 5b, 5BA and 9BA gelators form a single phase at the 1:1 ratio, (5BA)_1_(9BA)_1_ which shows lower *T*_m_^0^ at 127.0 °C compared to single 5BA and 9BA (Table S2).

#### 3.2.2 Even–Even Binary Gelators

The phase diagram of even–even binary systems (Figure 5c,d) follows the same trend as odd–odd gelators (Figure 5a,b): 6BA and 8BA form the compound (6BA)_1_(8BA)_1_ with very close melting points to the single 8BA gelators (Table S3). Similarly, (6BA)_1_(8BA)_1_ forms eutectic mixtures with 6BA and 8BA, respectively, at approximately 12.5% and 87.5% of 8BA in 6BA.

6BA and 10BA gelators mostly phase separate at 1:1, (6BA)_1_(10BA)_1_, melting at 138.7 °C, lower than single 6BA and 10BA (Table S4).

#### 3.2.3 Odd–Even Binary Gelators

Mixtures of 5BA with 6BA at the non-equimolar ratio of 3:1 show a dramatically lower melting temperature than single 5BA and 6BA gelators. The same trend is seen for (5BA)_3_(10BA)_1_ however with the presence of two different phases. The phase diagrams of these odd–even binary systems (Figure 5e,f) suggest that these two compositions still form eutectic mixtures.

An overview of the phase behavior in binary bisamides in the solid state has been given in Table 2.

**Table 2:** Molecular Assembly Behavior for Binary Bisamide Gelators in the Solid State, x Represents the Unstudied Mixtures

gelator	5BA	6BA	7BA	8BA	9BA	10BA
**5BA**	x	phase separation	compound	x	compound	phase separation
**6BA**		x	x	compound	x	mostly phase separation
**7BA**		x	x	x	x	x
**8BA**	x		x	x	x	x
**9BA**		x	x	x	x	x
**10BA**			x	x	x	x

### 3.3 Binary Bisamides in the Gel State

**Figure 6:**
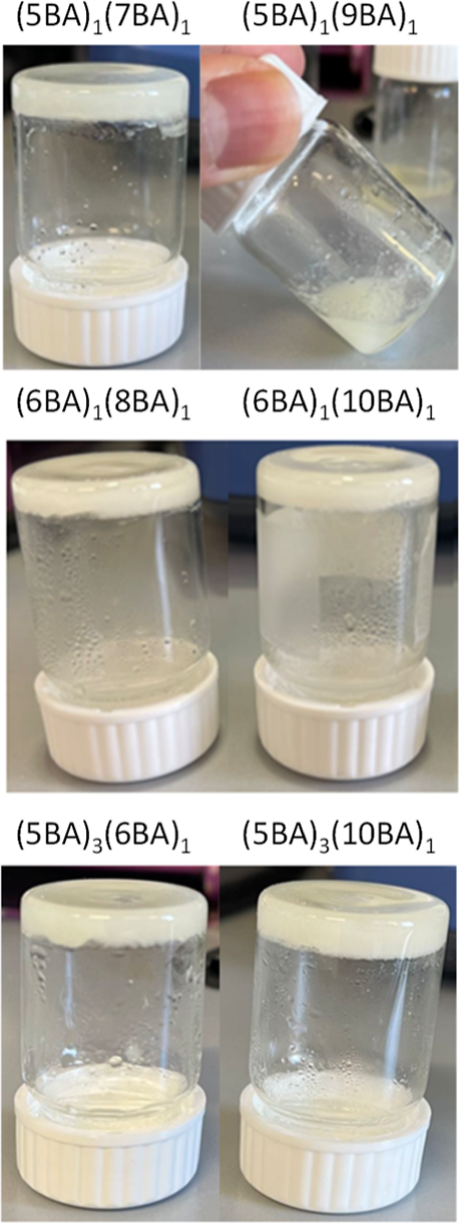
Binary gels (20 wt %) prepared from the eutectic mixtures and compound binary gelators.

To assess the gelation behavior of the binary compounds and mixtures compared to their single gelators, their gels were produced. To compare the characteristics of the binary gels with their single *n*BA gels from our previous study,^38^ all gels were prepared at 20 (wt %) concentration of the binary BAs under the same condition as single *n*BA gels (20 wt %). The gels made of binary gelators are called binary gels for simplicity. As assessed by the tube inversion test, all binary gels resisted flow by gravity except (5BA)_1_(9BA)_1_ (Figure 6).

To understand the assembly behavior of binary gelator molecules in the gel state, whether they mix (co-assemble) or phase separate (self-sort), the phase behavior and morphology of the binary gels were compared with their single *n*BA gels. The DSC thermograms of all *n*BA single gels and the underlying thermodynamics of their melting-dissolution transition were fully discussed in our previous study.^39^

#### 3.3.1 Odd Binary Gels

**Figure 7:**
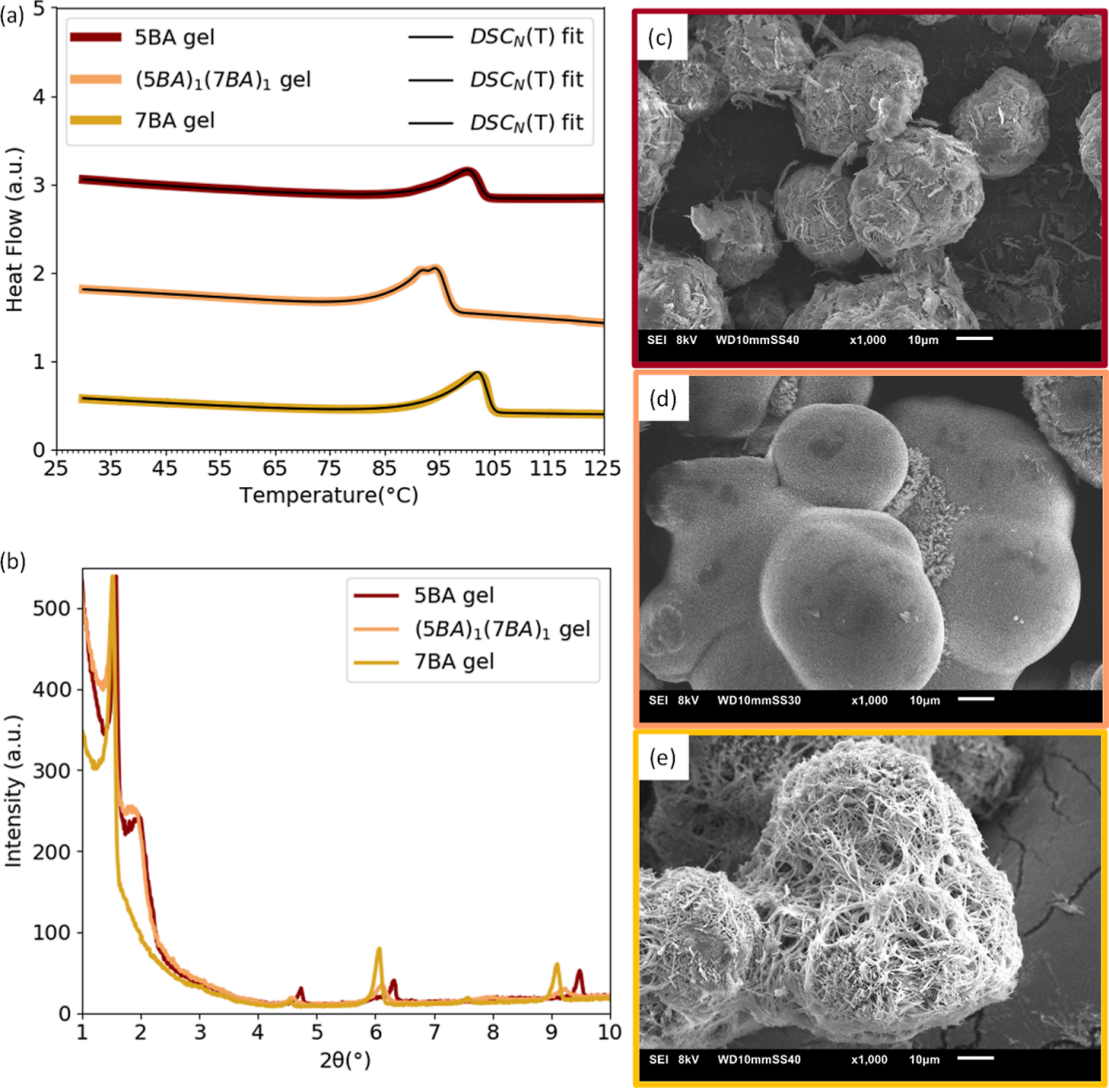
Phase behavior of 5BA, 7BA, and (5BA)_1_(7BA)_1_ gels (20 wt %): (a) DSC_N_(*T*) fits to the second heating traces (curves are shifted vertically for clarity), (b) diffraction patterns of single and binary 5BA7BA gels (curves were normalized to the highest intensity), SEM images of gels (20 wt %): (c) single 5BA, (d) (5BA)_1_(7BA)_1_, and (e) single 7BA at 1000× magnification.

The DSC thermogram of (5BA)_1_(7BA)_1_ gel (Figure 7a) shows a main melting-dissolution transitions with a minor residual transition on top where the *T*_m_^0^ is 10 °C lower than the *T*_m_^0^ of single 5BA gel and single 7BA gel (Table S7). To understand whether 5BA and 7BA molecules in (5BA)_1_(7BA)_1_ compounds have co-assembled or self-sorted in the gel state, the XRD pattern of (5BA)_1_(7BA)_1_ gel was compared with the respective single gels, as shown in Figure 7b. Similar to the low-angle XRD pattern of 5BA gel, the first-order reflection of (5BA)_1_(7BA)_1_ gel shows a clear splitting, where the first peak is at 1.52° (2θ), between the first-order reflection of 7BA gel and of 5BA gel, and the second peak is at 1.97° (2θ). Together with the fact that no further splitting is observed in any of the higher order reflections, as seen for 5BA gel, these observations indicate the tendency of these molecules for co-assembly. The microstructure of (5BA)_1_(7BA)_1_ gel in the SEM images (Figure 7d) displays common morphological features that this binary gel shares with single 5BA and 7BA gels;^38^ similar to single 5BA and 7BA gels, almost entirely a single morphology of spheres consisting of woven fibers is observed for (5BA)_1_(7BA)_1_ gel, however with much thinner fibers, which suggests the co-assembly of 5BA and 7BA molecules in the gel state. Co-assembly in the (5BA)_1_(7BA)_1_ gel state was observed in the solid state, where molecules formed the (5BA)_1_(7BA)_1_ compound. A small fraction of coarser fibers explains the minor DSC peak, which is assigned to the small deviation from the stoichiometric ratio.

The melting-dissolution of (5BA)_1_(9BA)_1_ gel occurs as a single phase transition (Figure S4a). Compared to single 5BA and 9BA gels, the melting-dissolution temperature of (5BA)_1_(9BA)_1_ gel shifts toward a lower temperature at 103.0 °C (Table S8). As shown in Figure S4b, none of the characteristic reflections in the low-angle XRD pattern of (5BA)_1_(9BA)_1_ gel shows splitting. Moreover, the first-order reflection of (5BA)_1_(9BA)_1_ gel appears at 1.40° (2θ), which is at a lower angle than that of 5BA gel [1.47° (2θ)] and 9BA gel [1.58° (2θ)]. In a reasonable agreement with the molecular arrangement in the solid state, 5BA and 9BA molecules, as shown in Figure S4d, have co-assembled into a woven fibrous structure in the (5BA)_1_(9BA)_1_ gel with a new distinct morphology from single 5BA gel and 9BA gel (Figure S4c,e).^38^

#### 3.3.2 Even Binary Gels

**Figure 8:**
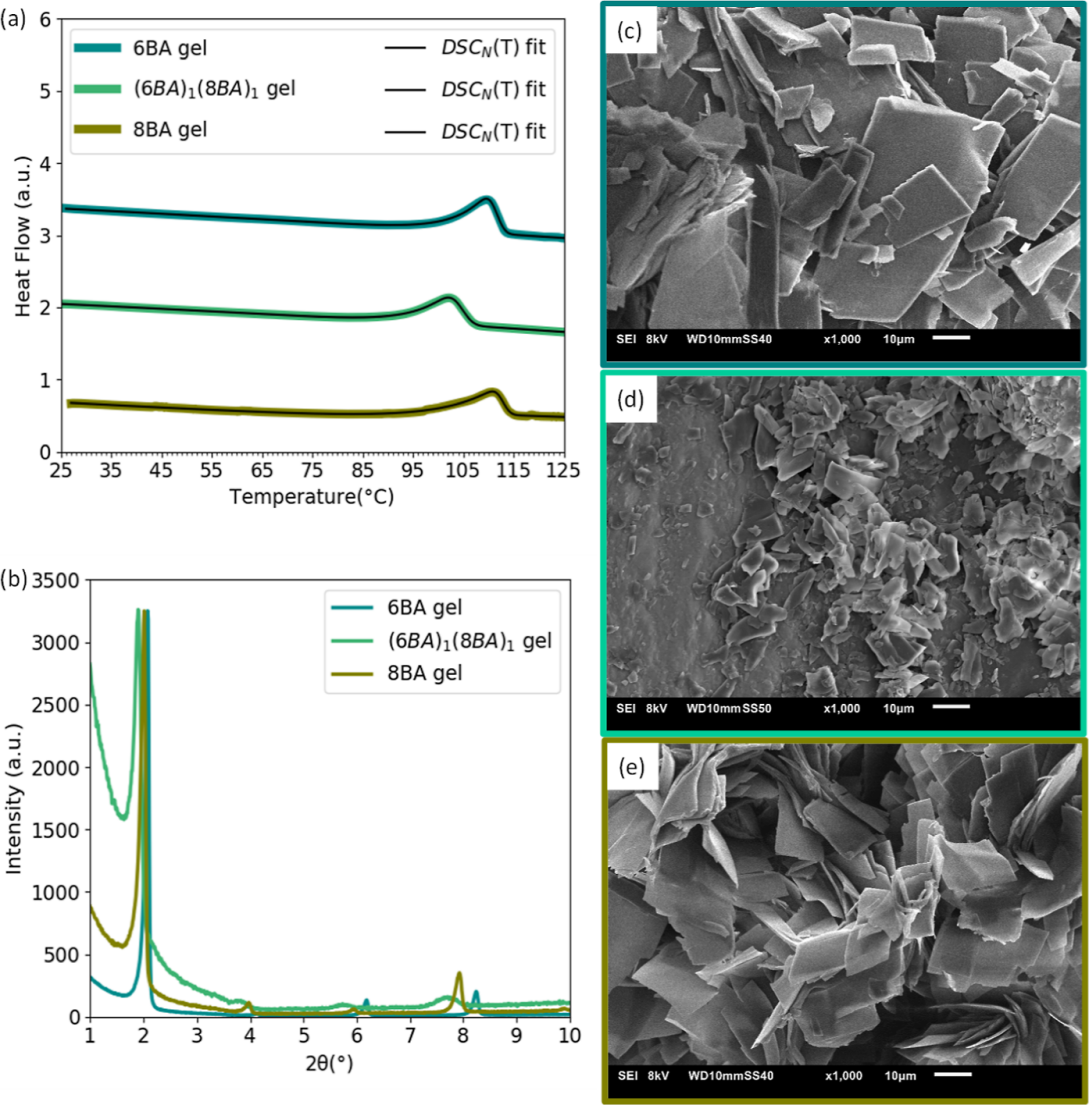
Phase behavior of 6BA, 8BA, and (6BA)_1_(8BA)_1_ gels (20 wt %): (a) DSC_N_(*T*) fits to the second heating traces (curves are shifted vertically for clarity), (b) diffraction patterns (curves were normalized to the highest intensity). SEM images of gels (20 wt %), (c) single 6BA, (d) (6BA)_1_(8BA)_1_ gel, and (e) single 8BA gel at 1000× magnification.

The melting-dissolution transition of the even–even binary gels from (6BA)_1_(8BA)_1_ and (6BA)_1_(10BA)_1_ are shifted toward lower temperatures compared to their single parent gels (Tables S9 and S10). The low-angle XRD pattern (Figure 8b) shows that the first-order reflection of (6BA)_1_(8BA)_1_ gel shifted to 1.91° (2θ), which is significantly lower than 6BA gel [2.07° (2θ)] and 8BA gels [2.01° (2θ)].

Similarly for (6BA)_1_(10BA)_1_ (Figure S5b), the first-order reflection shifted to the lower angle at 1.86° (2θ) compared to that of 6BA gel at 2.07° (2θ) and that of 10BA gel at 1.91° (2θ). However, a small wedge is observed at 1.99° (2θ), which can refer to a partial phase separation. Even–even gelator molecules co-assemble to form sheets in the (6BA)_1_(8BA)_1_ and (6BA)_1_(10BA)_1_ gels (Figure 8d and S5d), which is in line with the single melting-dissolution transition and the single first-order XRD reflection for (6BA)_1_(8BA)_1_ and (6BA)_1_(10BA)_1_ gels at different angles than their respective parent gels. However, the sheet-like structure of (6BA)_1_(8BA)_1_ is uniform than (6BA)_1_(10BA)_1_, where a mixture of sheets with a higher size distribution is observed. Investigating the molecular arrangement of single even gelators in our previous studies has shown that the even gelator molecules are tilted at an angle with respect to the layer normal^38^ and assemble into sheets in the gel state (Figure 8c,e and S5c,e).^39^ The shift toward the lower angles in the first-order reflection in the XRD pattern of binary gels with respect to that of single parent gels indicates the tilt is changing.

#### 3.3.3 Odd–Even Binary Gels

**Figure 9:**
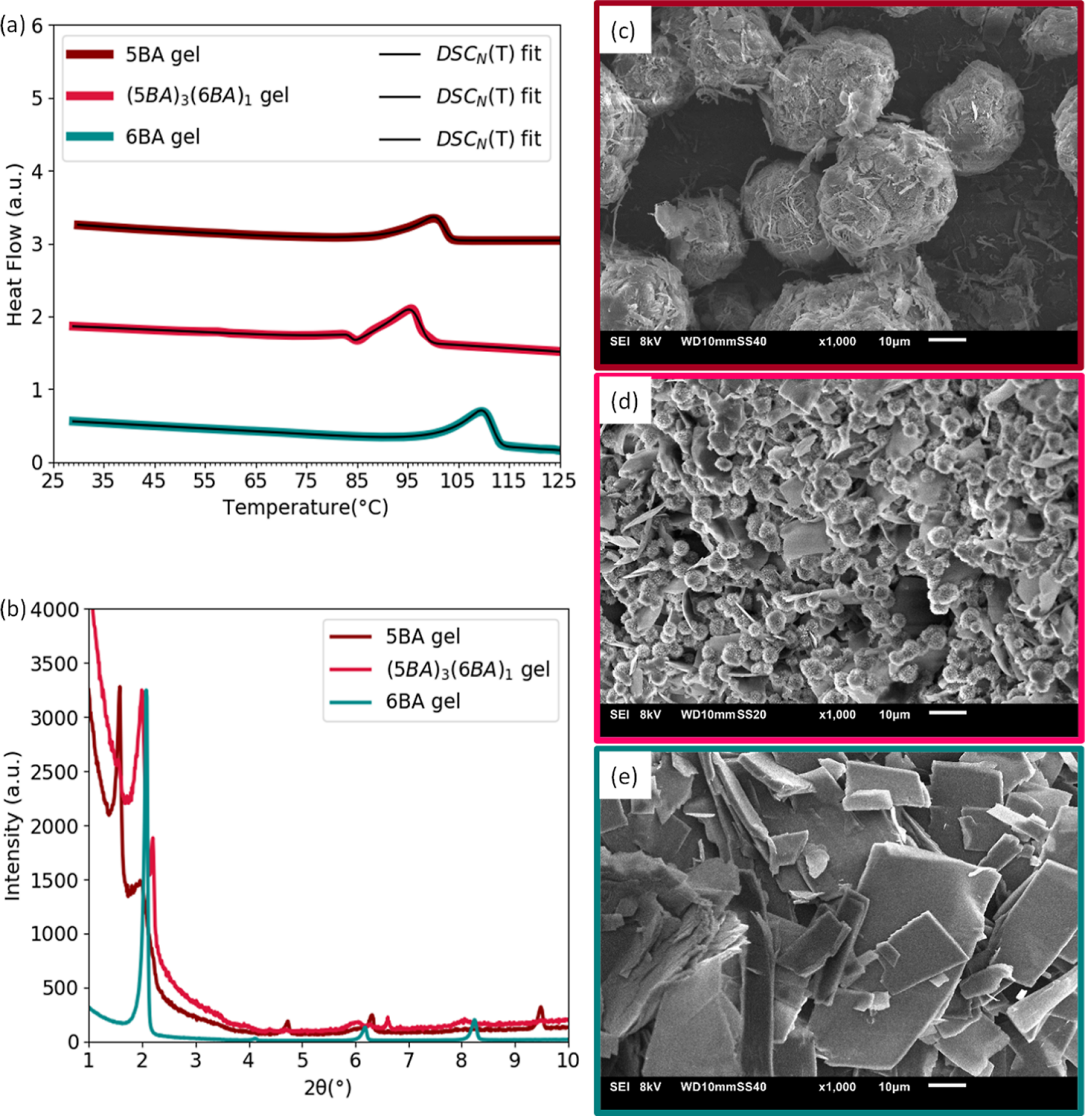
Phase behavior of 5BA, 6BA, and (5BA)_3_(6BA)_1_ gels (20 wt %): (a) DSC_N_(*T*) fits to the 2nd heating traces (curves are shifted vertically for clarity), (b) diffraction patterns (curves were normalized to the highest intensity). SEM images of gels (20 wt %), (c) single 5BA, (d) (5BA)_1_(6BA)_1_ gel, and (e) single 6BA gel at 1000× magnification.

The binary gel from (5BA)_3_(6BA)_1_ mixture shows a double melting-dissolution peak in the DSC thermogram in Figure 9a. Both peaks shifted to lower temperatures compared to the single peak of 5BA and 6BA gels (Table S11). The presence of two phases, likewise odd–odd and even–even binary gels, is evident from the split first-order and higher order reflections of (5BA)_3_(6BA)_1_ gel in their low-angle XRD patterns (Figure 9b). It is controversial to deduce a full self-sorting of 5BA and 6BA molecules in the binary gel because on the one hand the first-order reflection of 5BA gel is already split as discussed earlier. On the other hand, the split reflections in the XRD pattern of (5BA)_3_(6BA)_1_ gel do not identically correspond to the reflections of a single 5BA gel or to those of a 6BA gel. Despite this, the SEM images of (5BA)_3_(6BA)_1_ gel in Figure 9d show that the microstructure is the combination of woven sphere characteristic of a 5BA gel (Figure 9c) and sheets of a 6BA gel (Figure 9e). Altogether, these observations indicate that in the gel state these molecules tend to self-sort.

Comparable observations are made for (5BA)_3_(10BA)_1_ gel having a similar ratio as the (5BA)_3_(10BA)_1_ gelator (Figure S6). However, the low-angle XRD pattern of gel is the linear superposition of the patterns of single 5BA gel and 10BA gel, as seen in Figure S6b. Therefore, the self-sorting of 5BA and 10BA molecules in the (5BA)_3_(10BA)_1_ gel is more obvious than 5BA and 6BA in (5BA)_3_(6BA)_1_. This is probably due to the larger difference in their spacer length impeding their co-assembly via slight intermixing.

The assembly pattern of BA gelators in the gel state is summarized in Table 3.

**Table 3:** Assembly Behavior of Molecules in Binary Bisamides in the Gel State, x Represents the Unstudied Mixtures

Gel	5BA	6BA	7BA	8BA	9BA	10BA
5BA	x	self-sorting (sheet-fiber)	co-assembly (thin fiber)	x	co-assembly (woven fibers)	self-sorting (sheet-fiber)
6BA		x	x	co-assembly (sheet)	x	partial self-sorting (mixture of sheets)
7BA		x	x	x	x	x
8BA	x		x	x	x	x
9BA		x	x	x	x	x
10BA			x	x	x	x

### 3.4 Rheological Properties

To understand how the assembly behavior of molecules in the binary gels affects the rheological properties, the rheological properties of the binary gels are compared to those of their single parent gels, which were measured under the same conditions.^39^ For a more objective comparison, all gels were measured at the same concentration of 20 wt %, and the storage modulus (*G*′) of binary gels were compared with *G*′ of their parent *n*BA gels at a constant frequency (ω = 10 rad·s^–1^, which is in the linear viscoelastic region of all these gels at this concentration).

**Figure 10:**
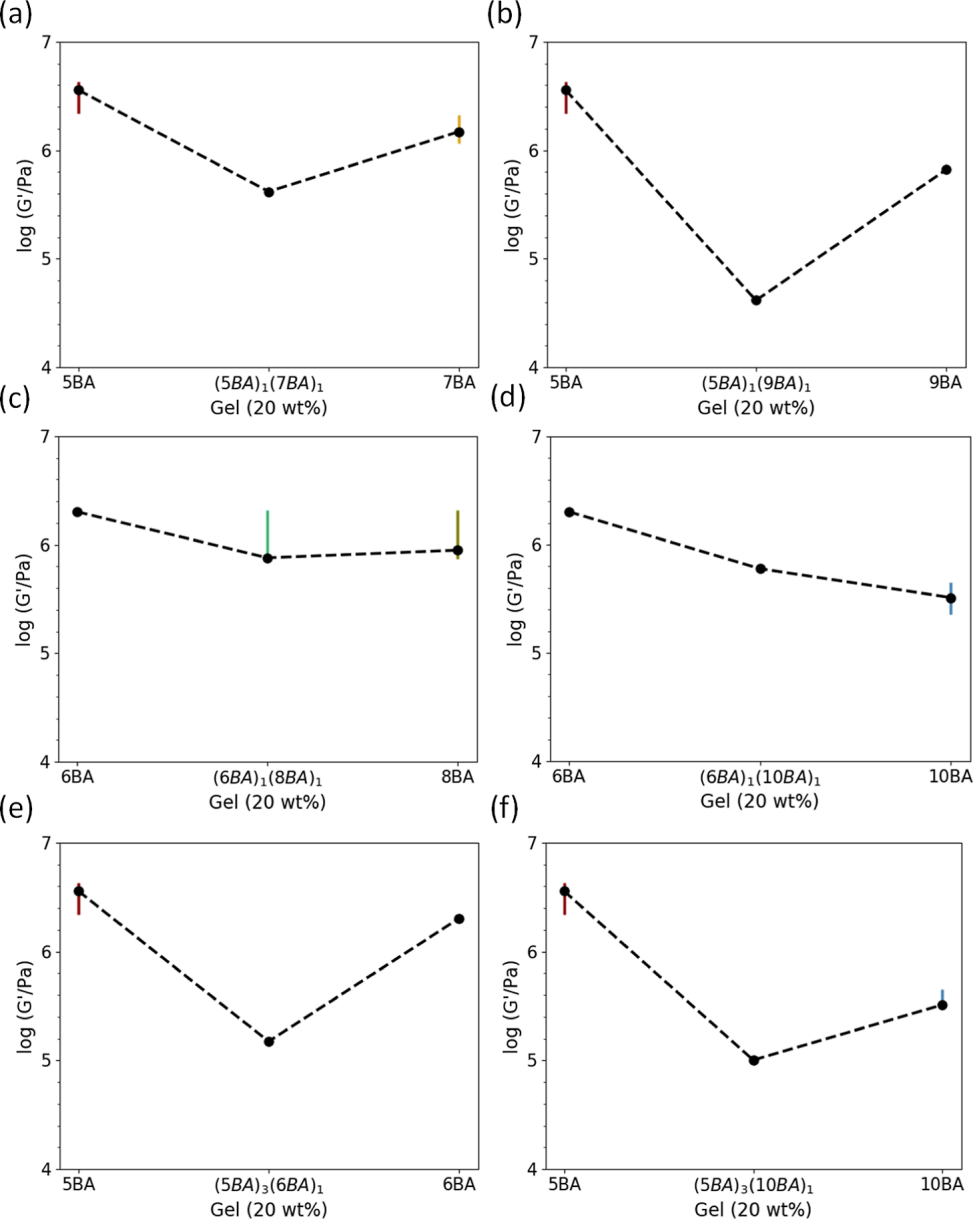
Storage modulus (*G*′) of binary gels (20 wt %) compared to their single parent gels (20 wt %) showing how the mechanical properties can be tailored by changing the second bisamide to the parent bisamide gelator: (a,b) odd–odd gels, (c,d) even–even gels, and (e,f) odd–even gels.

As shown in Figure 10a, the value of *G*′ for all binary gels is lower than their respective single parent gels except for (6BA)_1_(10BA)_1_, where *G*′ is approximately the average storage modulus of single 6BA and 10BA gels. Increasing the difference in the spacer length of the *n*BA components in both odd–odd (Figure 10a,b) and even–even (Figure 10c,d) binary gels has caused a more dramatic drop in the G′ of binary gels.

Co-assembly of even *n*BA molecules into sheets in even–even binary gels has endowed higher *G*′ to their gels than the co-assembly of odd molecules into fibrous structures in odd–odd binary gels (Table 2). The (5BA)_1_(9BA)_1_ gel has the lowest *G*′ among all binary gels, which is in line with the table-top inversion test (Table 4). The self-sorted gels with both sheets and woven spheres in their morphologies (Figures 9d and S6d) generally display a lower *G*′ than the co-assembled gels [except for the (5BA)_1_(9BA)_1_ gel]. Increasing the difference in the spacer length of the even–even BAs has decreased the *G*′ dramatically, which can be due to the presence of self-sorted 10BA molecules into sheets, which have shown the lowest *G*′ among all single *n*BA gels.^39^

**Table 4:** Effect of Assembly Behavior in Binary Gels (20 wt %) on their Storage Modulus (*G*′) Measured at the Certain Frequency [ω = 10 (rad·s^–1^)], x Represents the Unstudied Mixtures, the Data for Single Gels Are from Our Previous Study^39^

Gel	5BA	6BA	7BA	8BA	9BA	10BA
5BA	6.56 + 0.21/–0.08	5.22 + 0.04/–0.05	5.63 + 0.01/–0.01	x	4.63 + 0.02/–0.02	5.04 + 0.04/–0.04
	woven fibers and spherical structures	sheet-fibre	thin fibers		woven fibers	sheet-fiber
6BA		6.30 + 0.02/–0.02	x	6.32 + 0.44/-0.02	x	5.78 + 0.00/–0.00
		sheet		sheet		mixture of sheets
7BA		x	6.1 + 0.11/–0.16	x	x	x
			woven fibers			
8BA	x		x	5.95 + 0.08/–0.37	x	x
				sheet		
9BA		x	x	x	5.82 ± 0.00	x
					woven fibers	
10BA			x	x	x	5.51 + 0.15/–0.14
						sheet

## 4 Conclusions

This study on model binary *n*BA gelators aimed to establish design rules for binary mixture of gelators that govern the mixing or phase separation in the solid and gel states and investigate the effect of the phase behavior on the gel rheological properties.

From studying the arrangement of molecules in the solid state, the molecular self-recognition in binary bisamides with the same parity and close spacer lengths is not strong, which results in the formation of compounds [(5BA)_1_(7BA)_1_ and (6BA)_1_(8BA)_1_] with a close crystal structure and melting points to their single parent gelators. A larger difference in the spacer length leads to the partial phase separation of these molecules in (6BA)_1_(10BA)_1_. Although 5BA and 9BA are also four spacer lengths apart, these molecules still tend to mix in (5BA)_1_(9BA)_1_, which could be due to their unpaired hydrogen bonding pattern, giving them more freedom compared to (6BA)_1_(10BA)_1_. Different parities of BA molecules leads to the phase separation of these molecules in (5BA)_3_(6BA)_1_ and (5BA)_3_(10BA)_1_.

The gel architectures imply that in binary gels from binary compounds or partially mixed mixtures, molecules with the same parity co-assemble into fibers of the woven spheres (odd–odd gels) and into sheets (even–even gels). In binary gels from odd–even gelators, (5BA)_3_(6BA)_1_ and (5BA)_3_(10BA)_1_, the molecules self-sort to the separate woven spheres from sheets.

Comparing the supramolecular arrangement in the solid and gel states, the assembly pattern of molecules in the solid and gel states is the same for all binary bisamides; the solvent is indifferent to interaction with the molecules and does not change their assembly patterns.

Studying the rheological properties in correlation with morphological characteristics, all binary gels have lower *G*′ compared to their single parent gels except (6BA)_1_(10BA)_1_, where the *G*′ is nearly the average *G*′ of single 6BA and single 10BA gels. The decrease in the *G*′ of binary gels is more dramatic when the parity of the gelators is different or the difference in the spacer length increases. The rheological properties of the binary gels depend on the morphology of their gels, which can be primarily controlled using the odd–even symmetry of the molecules, where binary compounds or mixtures can lead to a variety of assembly behaviors: from co-assembly of even–even molecules into more homogeneous sheets to the co-assembly of odd–odd molecules into fibers in the woven spheres with less uniformity and ultimately the self-sorting of odd and even molecules into separate primary structures of spheres and sheets, which results in the least elastic gel behavior. In fact, although odd–odd also shows co-assembly, their microstructures are the combination of woven spheres, which apparently shows less entanglement than even–even sheets, and consequently lower storage modulus *G*′. It is in a reasonable agreement with the table-top inversion test where all binary gels (20 wt %) from eutectic mixtures and compounds could form stable gels with more flow resistance than the (5BA)_1_(9BA)_1_ gel (20 wt %).

## Supporting Information Available

Full details of phase behavior of binary systems and more analytical data from fitting of DSC_N_(*T*) model to the second heating traces of single and binary systems (PDF)
