# Supplementary material for: Design Rules for Binary Bisamide Gelators: toward Gels with Tailor-Made Structures and Properties
Source: Langmuir. 2023 Aug 14;39(34):12182–95. doi: 10.1021/acs.langmuir.3c01487 (PMC10469460; doi:10.1021/acs.langmuir.3c01487)
Supplement: Supplementary file 1 — la3c01487_si_001.pdf [file la3c01487_si_001.pdf]

Supporting Information
Design rules for binary bisamide gelators: toward gels with tailor-made structures and properties

Authors: Elmira Ghanbari[1], Stephen J. Picken[1], Jan H. van Esch[1,*]
[1]Advanced Soft Matter (ASM) group, Chemical engineering department, faculty of applied science (TNW), Delft University of Technology, 2629 HZ, Delft, The Netherlands
*Corresponding Author: Jan H. van Esch
Email: j.h.vanesch@tudelft.nl

Here, the phase behavior of binary gelators and gels and more analytical data from fitting of $DSC_N(T)$ model to the second heating traces of single and binary systems are provided:

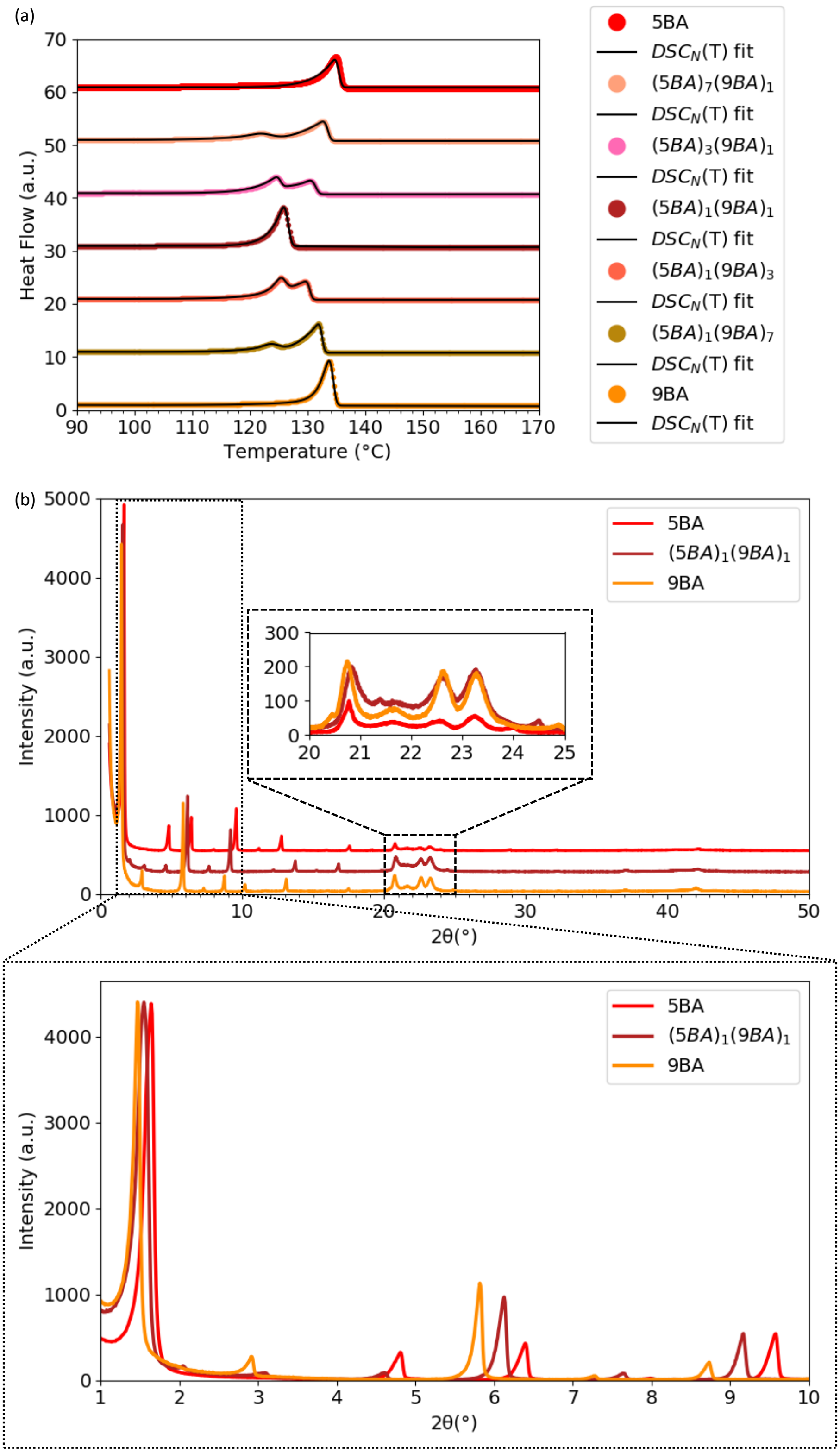

Figure S1. Phase behavior of 5BA9BA gelators: (a) DSC_N(T) fit to the second heating DSC traces of 5BA9BA at different mixing ratios (the traces and fits were shifted vertically for clarity) and (b) XRD patterns of $(5BA)_1(9BA)_1$ in comparison to single 5BA and 9BA gelators (curves were normalized to the highest intensity), the insets magnify high-angle (20°-25° (2Θ)) and low-angle (0°-10° (2Θ)) regions.

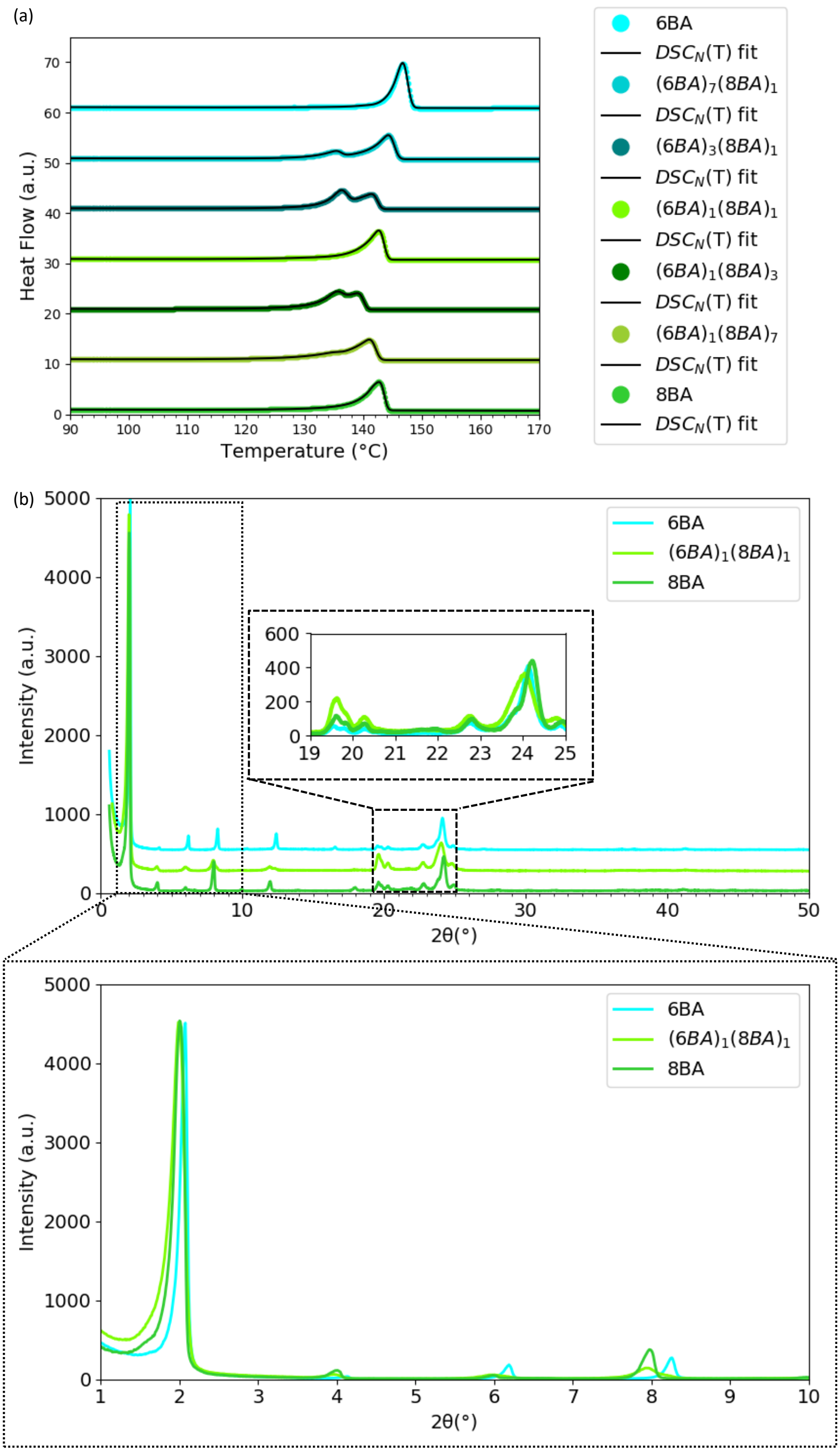

Figure S2. Phase behavior of 6BA8BA gelators: (a) the second heating traces for various mixing ratios 6BA and 8BA and $DSC_N(T)$ fits on the experimental traces (the traces and fits were shifted vertically for clarity) and (b) XRD patterns of $(6BA)_1(8BA)_1$ in comparison to single 6BA and 8BA gelators (curves were normalized to the highest intensity), the insets magnify high-angle (20°-25° (2ϴ)) and low-angle (0°-10° (2ϴ)) regions.

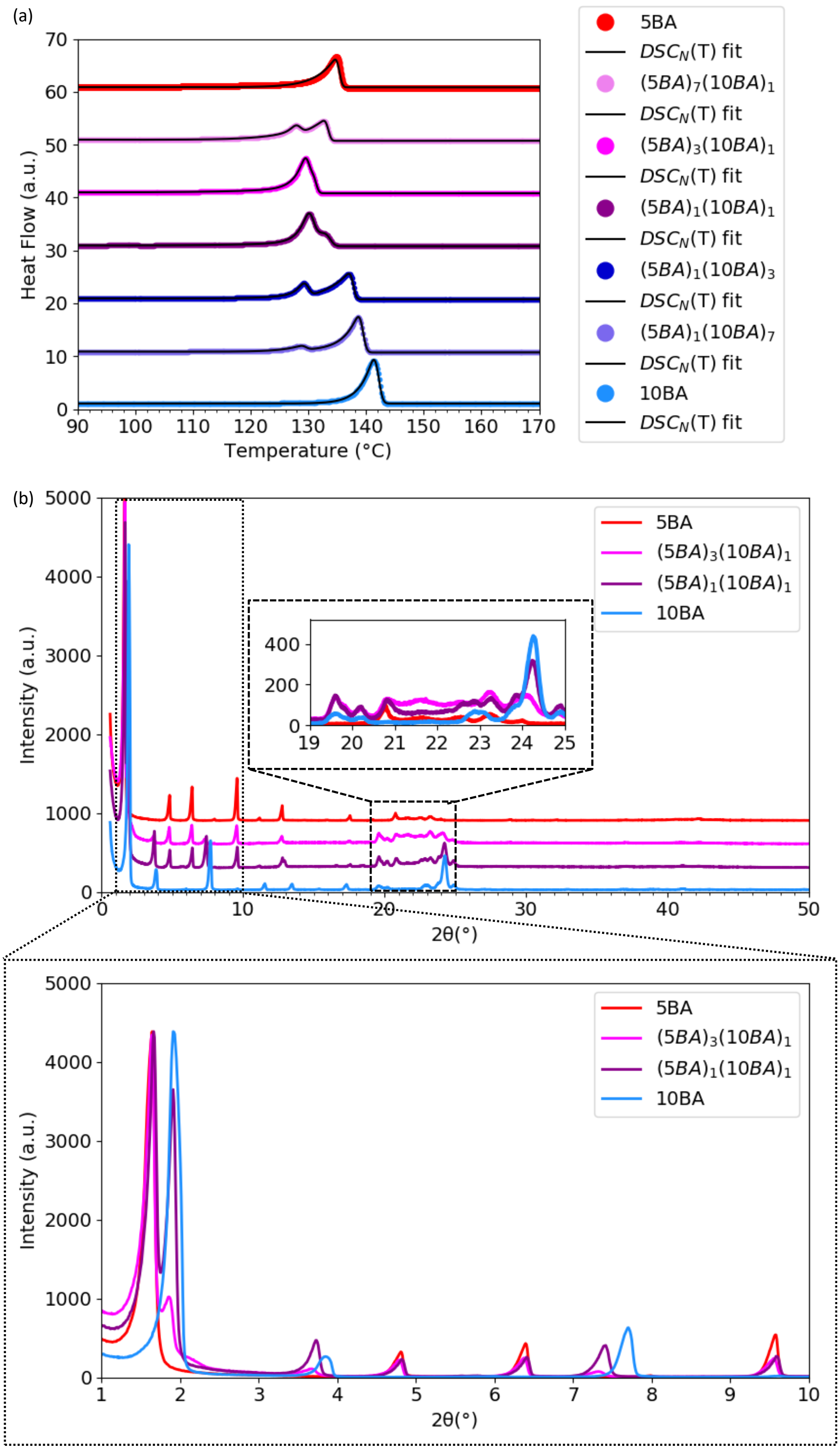

Figure S3. Phase behavior of 5BA10BA gelators: (a) the second heating traces for various mixing ratios 5BA and 10BA and $DSC_N(T)$ fits on the experimental traces (the traces and fits were shifted vertically for clarity) and (b) XRD patterns of $(5BA)_3(10BA)_1$ in comparison to single 5BA and 10BA gelators and binary $(5BA)_1(10BA)_1$ which also shows two DSC peaks and two distinct first-order reflections (curves were normalized to the highest intensity), the insets magnify high-angle (20°-25° (2Θ)) and low-angle (0°-10° (2Θ)) regions.

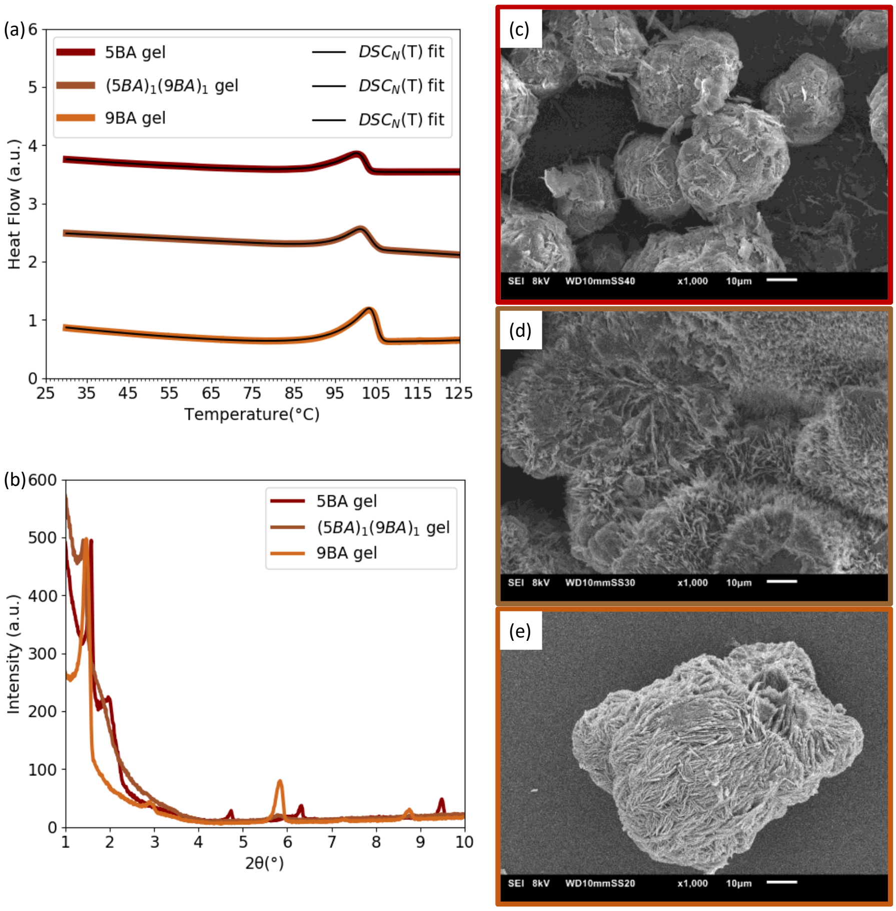

Figure S4. Phase behavior of 5BA, 9BA, and $(5BA)_1(9BA)_1$ gels (20 wt%): (a) DSC_N(T) fits to the second heating traces (curves are shifted vertically for clarity) and (b) diffraction patterns of 5BA, 9BA, and $(5BA)_1(9BA)_1$ gels (curves were normalized to the highest intensity). SEM images of gels (20 wt%) c) single 5BA, d) $(5BA)_1(9BA)_1$ gel, e) single 9BA gel at 1000x magnification.

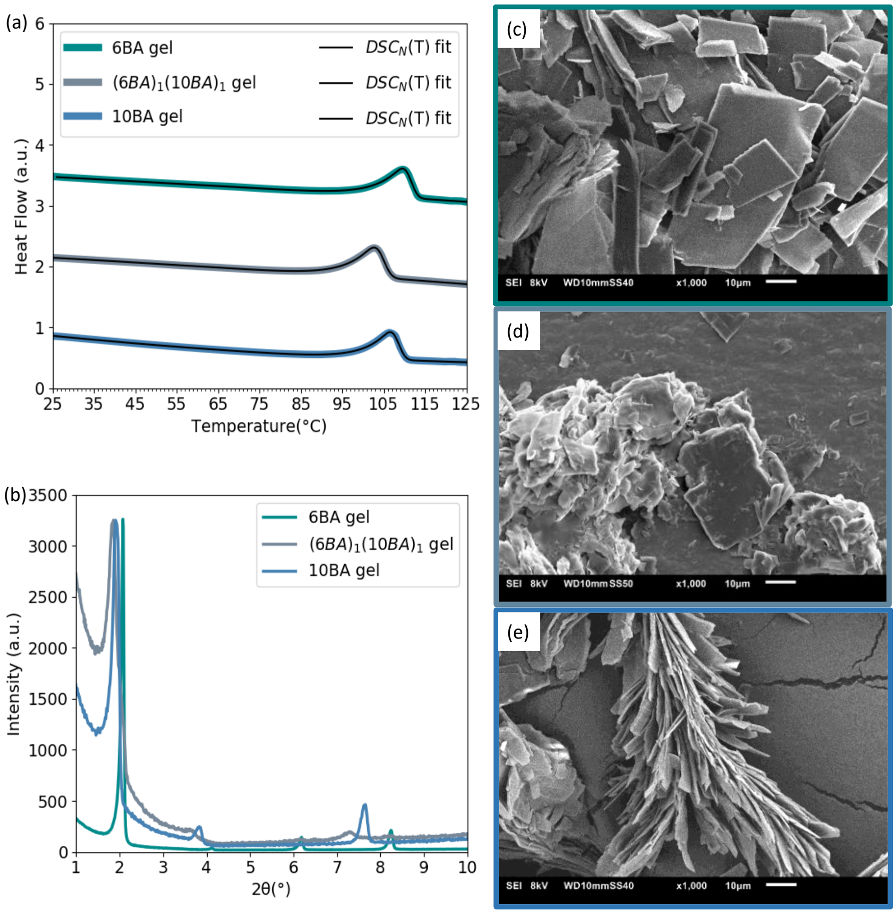

Figure S5. Phase behavior of 6BA, 10BA, and $(6BA)_1(10BA)_1$ gels (20 wt%): a) $DSC_N(T)$ fits to the second heating traces (curves are shifted vertically for clarity) and (b) diffraction patterns (curves were normalized to the highest intensity). SEM images of gels (20 wt%) c) single 6BA, d) $(6BA)_1(10BA)_1$ gel, e) single 10BA gel at 1000x magnification.

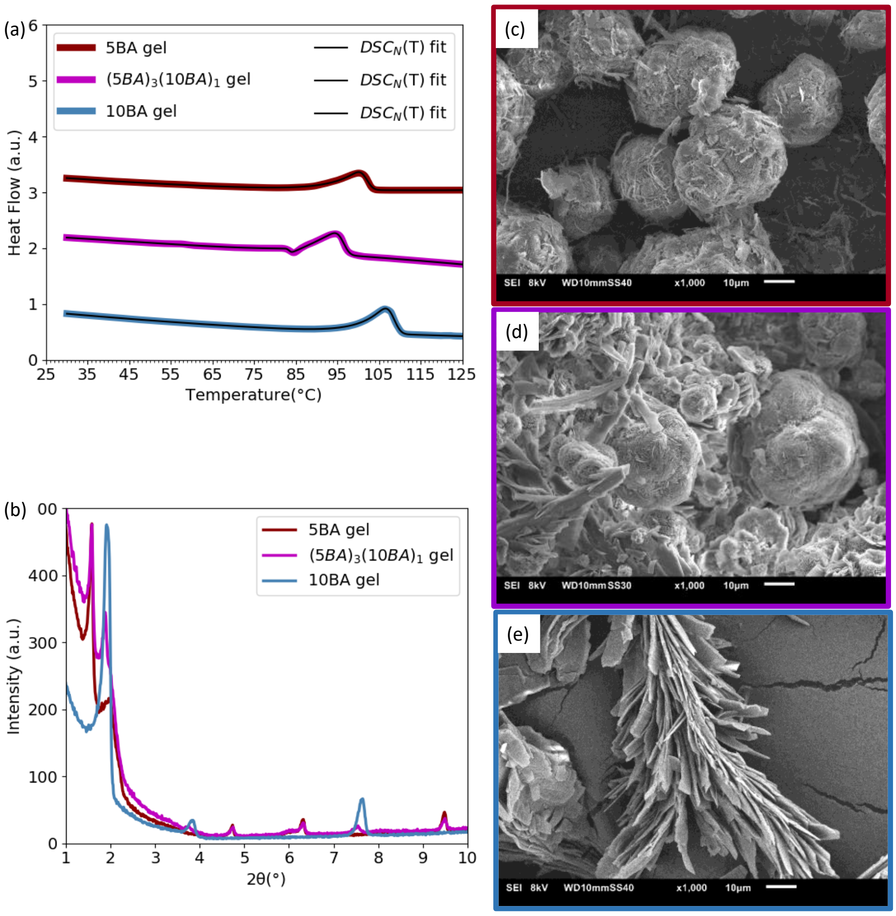

Figure S6. Phase behavior of 5BA, 10BA, and $(5BA)_3(10BA)_1$ gels (20 wt%): (a) $DSC_N(T)$ fits to the second heating traces (curves are shifted vertically for clarity) and (b) diffraction patterns(curves were normalized to the highest intensity). SEM images of gels (20 wt%) c) single 5BA, d) $(5BA)_1(10BA)_1$ gel, e) single 10BA gel at 1000x magnification.

Table S1. Fit parameters and statistical coefficient of the $DSC_N(T)$ function fitted to the experimental DSC trace of molecularly mixed binary 5BA7BA at different ratios [a].

Binary 5BA7BA	5BA	$(5BA)_1(7BA)_7$	$(5BA)_1(7BA)_3$	$(5BA)_1(7BA)_1$	$(5BA)_3(7BA)_1$	$(5BA)_7(7BA)_1$	7BA
First peak							
ΔH_1 ($J.g^{-1}$)		25.20±0.16	11.88±0.10		28.62±0.04	26.04±0.08	
$T_{m,1}^0$ (°C)	Single peak	124.60±0.05	125.09±0.06	Single peak	125.02±0.01	123.32±0.03	Single peak
α_1 (K^{-1})		0.19±0.00	0.64±0.05		0.59±0.01	0.17±0.00	
β_1 (K^{-2})		0.88±0.13	0.54±0.08		0.52±0.01	0.41±0.02	
$\Delta C_{p,m,1}$ ($W.g^{-1}.K^{-1}$)	NA						
Second peak							
ΔH_2 ($J.g^{-1}$)	126.18±0.04	104.52±0.23	116.64±0.29	131.82±0.12	120.96±0.09	82.98±0.13	0.09±140.88
$T_{m,2}^0$ (°C)	135.49±0.00	133.31±0.00	131.49±0.01	135.94±0.00	131.41±0.00	133.25±0.00	7±0.00135.9
α_2 (K^{-1})	0.39±0.00	0.39±0.01	0.19±0.00	0.59±0.00	0.18±0.00	0.30±0.00	0.00±0.52
β_2 (K^{-2})	3.01±0.04	1.84±0.04	2.47±0.09	1.41±0.01	1.88±0.02	1.76±0.02	1.50±0.03
$\Delta C_{p,m,2}$ ($W.g^{-1}.K^{-1}$)	-0.44±0.01	NA		-0.62±0.02	NA		-0.40±0.01

B ($W.g^{-1}$)	1.12±0.00	0.45±0.00	0.18±0.05	1.31±0.40	0.43±0.00	0.38±0.00	0.01±1.04
C ($mW.g^{1}.K^{-1}$)	0.00±0.00	-0.27±0.01	-0.01±0.00	-0.01±0.00	-0.13±0.03	-0.68±0.02	0.00±0.00
D ($mW.g^{-1}.K^{-2}$)	0.00±0.00	0.00±0.00	0.12±0.03	0.12±0.03	0.00±0.00	0.00±0.00	0.00±0.00
R^2	0.99	0.99	0.99	0.99	0.99	0.99	0.99

[a] The samples (6 mg) were heated at 10 (K min^{-1}) after calibration at the onset for the given weight and rate, in the case of $(5BA)_1(7BA)_1$, only one fitting peak is required due to the peak overlap (the error margins are from the nonlinear fitting), $\Delta C_{p,m}$ doesn't converge (Not available=NA) due to purely mathematical artefact, if the peaks are sufficiently apart with sufficient baseline tail on each side, the cumulative $\Delta C_{p,m}$ can be reliably determined via the $DSC_N(T)$ function for binary systems.

Table S2. Fit parameters and statistical coefficient of the $DSC_N(T)$ function fitted to the experimental DSC trace of 6mg of molecularly mixed binary 5BA9BA at different ratios [a].

Binary 5BA9BA	5BA	$(5BA)_1(9BA)_7$	$(5BA)_1(9BA)_3$	$(5BA)_1(9BA)_1$	$(5BA)_3(9BA)_1$	$(5BA)_7(9BA)_1$	9BA
First peak							
ΔH_1 ($J.g^{-1}$)	Single peak	18.06±0.09	39.78±0.03	Single peak	59.52±0.17	3.35±0.15	Single peak
$T_{m,1}^0$ (°C)		124.08±3.17	125.94±0.01		125.23±0.01	122.55±7.80	
α_1 (K^{-1})		9.66±300.42	0.74±0.01		0.27±0.00	8.3±0.80	
β_1 (K^{-2})		0.27±0.09	1.12±0.02		2.41±0.16	0.15±0.08	
$\Delta C_{p,m,1}$ ($W.g^{-1}.K^{-1}$)	NA						
Second peak							
ΔH_2 ($J.g^{-1}$)	126.18±0.04	189.06±0.13	138.78±0.08	150.12±0.14	49.26±0.83	184.44±0.18	149.21±0.08
$T_{m,2}^0$ (°C)	135.49±0.00	132.47±0.00	130.33±0.00	126.54±0.00	131.31±0.015	133.44±0.01	132.50±0.00
α_2 (K^{-1})	0.39±0.00	0.21±0.00	0.17±0.00	0.44±0.00	0.41±0.05	0.14±0.00	0.52±0.00
β_2 (K^{-2})	3.01±0.04	3.91±0.07	3.46±0.06	2.02±0.04	1.80±0.10	2.46±0.07	2.68±0.05
$\Delta C_{p,m,2}$ ($W.g^{-1}.K^{-1}$)	-0.44±0.01	NA		0.00+0.04	NA		-0.35±0.01
B ($W.g^{-1}$)	1.12±0.00	0.45±0.00	0.44±0.00	0.46±0.26	0.45±0.00	0.443±0.00	1.07±0.01
C ($mW.g^{1}.K^{-1}$)	0.00±0.00	-0.365±0.01	-0.43±0.02	1.32±0.08	0.02±0.06	-0.41±0.00	0.00±0.00
D ($mW.g^{-1}.K^{-2}$)	0.00±0.00	0.01±0.00	0.01±0.00	0.02±0.00	0.01±0.00	0.01±0.00	0.00±0.00
R^2	0.99	0.99	0.999	0.996	0.995	0.996	0.99

[a] The samples (6 mg) were heated at 10 (K min^{-1}) after calibration at the onset for the given weight and rate, in the case of $(5BA)_1(9BA)_1$, only one fitting peak is required due to the peak overlap (the error margins are from the nonlinear fitting), $\Delta C_{p,m}$ does not converge (Not available=NA) due to purely mathematical artefact, if the peaks are sufficiently apart with sufficient baseline tail on each side, the cumulative $\Delta C_{p,m}$ can be reliably determined via the $DSC_N(T)$ function for binary systems.

Table S3. Fit parameters and statistical coefficient of the $DSC_N(T)$ function fitted to the experimental DSC trace of 6mg of molecularly mixed binary 6BA8BA at different ratios[a].

Binary 6BA8BA	6BA	$(6BA)_1(8BA)_7$	$(6BA)_1(8BA)_3$	$(6BA)_1(8BA)_1$	$(6BA)_3(8BA)_1$	$(6BA)_7(8BA)_1$	8BA
First peak							
ΔH_1 ($J.g^{-1}$)	Single peak	36.96±1.05	34.02±0.09	Single peak	49.38±0.05	32.46±0.07	Single peak
$T_{m,1}^0$ (°C)		135.68±0.06	136.18±0.02		137.04±0.01	136.20±0.02	
α_1 (K^{-1})		0.09±0.01	0.50±0.01		0.59±0.01	0.28±0.00	
β_1 (K^{-2})		0.71±0.12	0.84±0.05		0.88±0.02	1.28±0.07	
$\Delta C_{p,m,1}$ ($W.g^{-1}.K^{-1}$)	NA						
Second peak							
ΔH_2 ($J.g^{-1}$)	168.66±0.07	142.26±1.14	149.04±0.29	162.30±1.91	127.02±0.11	120.66±0.18	155.12±0.12
$T_{m,2}^0$ (°C)	147.46±0.00	142.09±0.01	140.09±0.01	143.51±0.00	142.42±0.00	145.24±0.00	143.53±0.00
α_2 (K^{-1})	0.63±0.00	0.23±0.01	0.15±0.00	0.30±0.02	0.18±0.00	0.36±0.00	0.31±0.00
β_2 (K^{-2})	1.19±0.02	1.24±0.02	1.77±0.05	1.67±0.04	1.61±0.03	1.14±0.02	1.69±0.04
$\Delta C_{p,m,2}$ ($W.g^{-1}.K^{-1}$)	-0.28±0.01	NA		0.27±0.52	NA		-0.28±0.01
B ($W.g^{-1}$)	1.12±0.01	0.42±0.00	0.44±0.00	0.38±0.00	0.44±0.00	0.43±0.00	0.94±0.01
C ($mW.g^{-1}.K^{-1}$)	0.00±0.00	-0.29±0.06	-0.25±0.05	-0.26±0.09	0.03±0.04	0.31±0.05	0.00±0.00
D ($mW.g^{-1}.K^{-2}$)	0.00±0.00	0.01±0.00	0.01±0.00	0.01±0.00	0.01±0.00	0.01±0.00	0.00±0.00
R^2	0.99	0.99	0.99	0.99	0.99	0.99	0.99

[a] The samples (6 mg) were heated at 10 ($K\ min^{-1}$) after calibration at the onset for the given weight and rate, in the case of $(6BA)_1(8BA)_1$, only one fitting peak is required due to the peak overlap (the error margins are from the nonlinear fitting), $\Delta C_{p,m}$ does not converge (Not available=NA) due to purely mathematical artefact, if the peaks are sufficiently apart with sufficient baseline tail on each side, the cumulative $\Delta C_{p,m}$ can be reliably determined via the $DSC_N(T)$ function for binary systems.

Table S4. Fit parameters and statistical coefficient of the $DSC_N(T)$ function fitted to the experimental DSC trace of 6mg of molecularly mixed binary 6BA10BA at different ratios[a].

**Binary 6BA10BA**	**6BA**	**$(6BA)_1(10BA)_7$**	**$(6BA)_1(10BA)_3$**	**$(6BA)_1(10BA)_1$**	**$(6BA)_3(10BA)_1$**	**$(6BA)_7(10BA)_1$**	**10BA**
First peak							
ΔH_1 (J.g^{-1})	Single peak	34.56±0.13	49.32±0.03	125.16±0.07	58.56±0.04	31.74±0.04	Single peak
$T_{m,1}^0$ (°C)		135.26±0.01	135.74±0.00	136.35±0.00	135.22±0.00	134.26±0.01	
α_1 (K^{-1})		1.21±0.00	0.84±0.00	0.73±0.00	0.80±0.01	0.41±0.00	
β_1 (K^{-2})		1.85±0.15	1.81±0.02	1.21±0.01	1.64±0.02	3.24±0.15	
$\Delta C_{p,m,1}$ (W.g^{-1}.K^{-1})	NA						
Second peak							
ΔH_2 (J.g^{-1})	168.66±0.07	148.38±0.01	310.08±1.17	164.52±1.49	136.98±0.14	122.40±0.07	172.20±0.06
$T_{m,2}^0$ (°C)	147.46±0.00	138.89±0.07	138.02±0.00	138.71±0.01	143.33±0.00	145.40±0.00	142.11±0.00
α_2 (K^{-1})	0.63±0.00	0.35±0.07	0.12±0.00	0.08±0.00	0.20±0.00	0.40±0.00	0.48±0.00
β_2 (K^{-2})	1.19±0.02	2.22±0.04	3.45±0.02	1.50±0.06	2.62±0.05	2.21±0.03	1.40±0.02
$\Delta C_{p,m,2}$ (W.g^{-1}.K^{-1})	-0.28±0.01	NA		-1.72+0.03	NA		-0.29±0.01
B (W.g^{-1})	1.12±0.01	0.46±0.00	0.62±0.00	0.39±0.00	0.00±0.00	0.44±0.00	1.19±0.01
C (mW.g^{-1}.K^{-1})	0.00±0.00	0.38±5.08	1.63±0.02	-0.39±0.04	-0.14±0.05	0.29±0.05	0.01±0.00
D (mW.g^{-1}.K^{-2})	0.00±0.00	0.01±0.00	0.01±0.00	0.01±0.00	0.01±0.00	0.01±0.00	0.00±0.00
R^2	0.99	0.99	0.99	0.99	0.99	0.99	0.99

[a] The samples (6 mg) were heated at 10 (K min^{-1}) after calibration at the onset for the given weight and rate, in the case of $(6BA)_1(10BA)_1$, only one fitting peak is required due to the peak overlap (the error margins are from the nonlinear fitting), $\Delta C_{p,m}$ does not converge (Not available=NA) due to purely mathematical artefact, if the peaks are sufficiently apart with sufficient baseline tail on each side, the cumulative $\Delta C_{p,m}$ can be reliably determined via the $DSC_N(T)$ function for binary systems.

Table S5. Fit parameters and statistical coefficient of the $DSC_N(T)$ function fitted to the experimental DSC trace of 6mg of molecularly mixed binary 5BA6BA at different ratios[a].

Binary 5BA6BA	5BA	$(5BA)_1(6BA)_7$	$(5BA)_1(6BA)_3$	$(5BA)_1(6BA)_1$	$(5BA)_3(6BA)_1$	$(5BA)_7(6BA)_1$	6BA
First peak							
ΔH_1 $(J.g^{-1})$	Single peak	22.08±0.05	37.56±0.03	69.54±0.02	Single peak	56.34±0.76	Single peak
$T_{m,1}^0$ (°C)		130.49±0.03	130.59±0.00	131.49±0.00		129.00±0.02	
α_1 (K^{-1})		0.17±0.00	0.38±0.00	0.61±0.00		0.07±0.00	
β_1 (K^{-2})		1.20±0.12	1.41±0.04	1.94±0.02		1.62±0.21	
$\Delta C_{p,m,1}$ $(W.g^{-1}.K^{-1})$	NA						
Second peak							
ΔH_2 $(J.g^{-1})$	126.18±0.04	127.44±0.06	107.76±0.11	115.02±0.12	222.3±7.07	120.6±0.23	168.66±0.07
$T_{m,2}^0$ (°C)	135.49±0.00	145.46±0.00	142.97±0.00	137.94±0.01	132.21±0.02	133.62±0.00	147.46±0.00
α_2 (K^{-1})	0.39±0.00	0.36±0.00	0.27±0.00	0.09±0.00	0.07±0.00	0.30±0.00	0.63±0.00
β_2 (K^{-2})	3.01±0.04	1.50±0.02	1.89±0.03	1.11±0.02	2.43±0.28	2.65±0.04	1.19±0.02
$\Delta C_{p,m,2}$ $(W.g^{-1}.K^{-1})$	-0.44±0.01	NA			-1.85±0.52	NA	-0.28±0.01
B $(W.g^{-1})$	1.12±0.00	0.38±0.00	0.51±0.00	0.38±0.00	0.38±0.00	0.39±0.00	1.12±0.01
C $(mW.g^{-1}.K^{-1})$	0.00±0.00	-0.24±0.05	0.64±0.04	-0.45±0.03	-0.59±0.02	-0.54±0.04	0.00±0.00
D $(mW.g^{-1}.K^{-2})$	0.00±0.00	0.01±0.00	0.01±0.00	0.01±0.00	0.01±0.00	0.01±0.00	0.00±0.00
R^2	0.99	0.99	0.99	0.99	0.99	0.99	0.99

[a] The samples (6 mg) were heated at 10 (K min^{-1}) after calibration at the onset for the given weight and rate, in the case of $(5BA)_3(6BA)_1$, only one fitting peak is required due to the peak overlap (the error margins are from the nonlinear fitting), $\Delta C_{p,m}$ does not converge (Not available=NA) due to purely mathematical artefact, if the peaks are sufficiently apart with sufficient baseline tail on each side, the cumulative $\Delta C_{p,m}$ can be reliably determined via the $DSC_N(T)$ function for binary systems.

Table S6. Fit parameters and statistical coefficient of the $DSC_N(T)$ function fitted to the experimental DSC trace of 6mg of molecularly mixed binary 5BA10BA at different ratios[a].

Binary 5BA10BA	5BA	$(5BA)_1(10BA)_7$	$(5BA)_1(10BA)_3$	$(5BA)_1(10BA)_1$	$(5BA)_3(10BA)_1$	$(5BA)_7(10BA)_1$	10BA
First peak							
ΔH_1 ($J.g^{-1}$)	Single peak	22.62±0.00	33.36±0.05	76.38±0.03	62.28±0.14	18.12±0.06	Single peak
$T_{m,1}^0$ (°C)		0.00±0.00	129.87±0.01	130.71±0.00	129.98±0.01	128.44±0.02	
α_1 (K^{-1})		0.00±0.00	0.85±0.02	0.64±0.00	0.60±0.00	1.21±0.08	
β_1 (K^{-2})		0.99	1.41±0.04	1.29±0.01	2.08±0.05	1.16±0.07	
$\Delta C_{p,m,1}$ ($W.g^{-1}.K^{-1}$)	NA						
Second peak							
ΔH_2 ($J.g^{-1}$)	126.18±0.04	24.57±0.07	169.32±0.13	169.26±0.40	250.98±0.90	147.30±0.09	172.20±0.06
$T_{m,2}^0$ (°C)	135.49±0.00	139.42±0.00	137.76±0.00	133.78±0.00	131.33±0.01	133.42±0.00	142.11±0.00
α_2 (K^{-1})	0.39±0.00	0.40±0.01	0.21±0.00	0.11±0.00	0.09±0.00	0.19±0.00	0.48±0.00
β_2 (K^{-2})	3.01±0.04	1.87±0.01	2.87±0.04	1.17±0.02	3.03±0.08	2.80±0.07	1.40±0.02
$\Delta C_{p,m,2}$ ($W.g^{-1}.K^{-1}$)	-0.44±0.01	NA			-2.61±0.05	NA	-0.29±0.01
B ($W.g^{-1}$)	1.12±0.00	0.42±0.00	0.44±0.00	0.43±0.00	0.36±0.00	0.58±0.03	1.19±0.01
C ($mW.g^{-1}.K^{-1}$)	0.00±0.00	-0.15±0.03	-0.11±0.04	-0.47±0.00	-0.46±0.02	-0.12±0.04	0.01±0.00
D ($mW.g^{-1}.K^{-2}$)	0.00±0.00	0.01±0.00	0.01±0.00	1.44±1.91	0.01±0.00	0.01±0.00	0.00±0.00
R^2	0.99	0.99	0.99	0.99	0.99	0.99	0.99

[a] The samples (6 mg) were heated at 10 ($K\ min^{-1}$) after calibration at the onset for the given weight and rate, in the case of $(5BA)_3(10BA)_1$, only one fitting peak is required due to the peak overlap (the error margins are from the nonlinear fitting), $\Delta C_{p,m}$ does not converge (Not available=NA) due to purely mathematical artefact, if the peaks are sufficiently apart with sufficient baseline tail on each side, the cumulative $\Delta C_{p,m}$ can be reliably determined via the $DSC_N(T)$ function for binary systems.

Table S7. Fit parameters and statistical coefficient of the $DSC_N(T)$ function fitted to the experimental DSC trace of 8mg of 5BA, 7BA, and $(5BA)_1(7BA)_1$ gels (20 wt%)[a].

**5BA7BA gels (20 wt%)**	**5BA gel**	**$(5BA)_1(7BA)_1$ gel**	**7BA gel**
ΔH_1 ($J.g^{-1}$)	Single peak	93.36±0.01	Single peak
$T_{m,1}^0$ (°C)		92.51±0.01	
α_1 (K^{-1})		0.00±0.00	
β_1 (K^{-2})		1.45±0.07	
$\Delta C_{p,m,1}$ ($W.g^{-1}.K^{-1}$)		NA	
ΔH_2 ($J.g^{-1}$)	36.12±0.01	59.64±0.02	53.16±0.01
$T_{m,2}^0$ (°C)	101.96±0.01	95.96±0.00	103.63±0.00
α_2 (K^{-1})	0.15±0.00	0.15±0.00	0.14±0.00
β_2 (K^{-2})	0.33±0.00	0.34±0.00	0.52±0.00
$\Delta C_{p,m,2}$ ($W.g^{-1}.K^{-1}$)	0.01±0.00	NA	0.03±0.00
B ($W.g^{-1}$)	0.54±0.00	5.05±1.74	0.39±0.00
C ($mW.g^{-1}.K^{-1}$)	-0.86±0.01	-1.92±0.03	-1.30±0.02
D ($mW.g^{-1}.K^{-2}$)	0.03±0.00	-0.01±0.00	0.02±0.00
R^2	0.99	0.99	0.99

[a] The samples were heated at 5 (K min^{-1}) after calibration at the onset for the given weight and rate, in the case of single gels only one fitting peak is required (the error margins are from the nonlinear fitting), $\Delta C_{p,m}$ does not converge (Not available=NA) due to purely mathematical artefact, if the peaks are sufficiently apart with sufficient baseline tail on each side, the cumulative $\Delta C_{p,m}$ can be reliably determined via the $DSC_N(T)$ function for binary systems.

Table S8. Fit parameters and statistical coefficient of the $DSC_N(T)$ function fitted to the experimental DSC trace of 8mg of 5BA, 9BA, and $(5BA)_1(9BA)_1$ gels (20 wt%)[a].

**5BA9BA gels (20 wt%)**	**5BA gel**	**$(5BA)_1(9BA)_1$ gel**	**9BA gel**
ΔH_1 ($J.g^{-1}$)	36.12±0.01	37.56±0.00	64.08±0.00
$T_{m,1}^0$ (°C)	101.96±0.01	103.13±0.00	104.82±0.00
α_1 (K^{-1})	0.15±0.00	0.18±0.00	0.15±0.00
β_1 (K^{-2})	0.33±0.00	0.19±0.00	0.44±0.00
$\Delta C_{p,m,1}$ ($W.g^{-1}.K^{-1}$)	0.01±0.00	0.08+0.00	0.04±0.00
B ($W.g^{-1}$)	0.54±0.00	0.20±0.00	1.59±0.00
C ($mW.g^{-1}.K^{-1}$)	-0.86±0.01	-4.20±0.01	1.02±0.01
D ($mW.g^{-1}.K^{-2}$)	0.03±0.00	-0.01±0.00	0.05±0.00
R^2	0.99	0.99	0.99

[a] The samples were heated at 5 (K min^{-1}) after calibration at the onset for the given weight and rate, in the case of single gels only one fitting peak is required (the error margins are from the nonlinear fitting), $\Delta C_{p,m}$ does not converge (Not available=NA) due to purely mathematical artefact, if the peaks are sufficiently apart with sufficient baseline tail on each side, the cumulative $\Delta C_{p,m}$ can be reliably determined via the $DSC_N(T)$ function for binary systems.

Table S9. Fit parameters and statistical coefficient of the $DSC_N(T)$ function fitted to the experimental DSC trace of 8mg of 6BA, 8BA, and $(6BA)_1(8BA)_1$ gels (20 wt%)[a].

6BA8BA gels (20 wt%)	6BA gel	$(6BA)_1(8BA)_1$ gel	8BA gel
ΔH_1 ($J.g^{-1}$)	53.04±0.01	49.56±0.00	53.88±0.01
$T_{m,1}^0$ (°C)	111.50±0.00	104.51±0.00	112.77±0.00
α_1 (K^{-1})	0.16±0.00	0.15±0.00	0.11±0.00
β_1 (K^{-2})	0.28±0.00	0.15±0.00	0.33±0.00
$\Delta C_{p,m,1}$ ($W.g^{-1}.K^{-1}$)	0.00±0.00	0.01+0.00	0.01±0.00
B ($W.g^{-1}$)	0.22±0.00	0.24±0.00	0.41±0.00
C ($mW.g^{-1}.K^{-1}$)	-4.45±0.03	-4.49±0.01	-2.48±0.02
D ($mW.g^{-1}.K^{-2}$)	0.01±0.00	-0.01±0.00	0.01±0.00
R^2	0.99	0.99	0.99

[a] The samples were heated at 5 ($K\ min^{-1}$) after calibration at the onset for the given weight and rate, in the case of single gels only one fitting peak is required (the error margins are from the nonlinear fitting), $\Delta C_{p,m}$ does not converge (Not available=NA) due to purely mathematical artefact, if the peaks are sufficiently apart with sufficient baseline tail on each side, the cumulative $\Delta C_{p,m}$ can be reliably determined via the $DSC_N(T)$ function for binary systems.

Table S10. Fit parameters and statistical coefficient of the $DSC_N(T)$ function fitted to the experimental DSC trace of 8mg of 6BA, 10BA, and $(6BA)_1(10BA)_1$ gels (20 wt%)[a].

6BA10BA gels (20 wt%)	6BA gel	$(6BA)_1(10BA)_1$ gel	10BA gel
ΔH_1 ($J.g^{-1}$)	53.04±0.01	59.16±0.00	47.88±0.01
$T_{m,1}^0$ (°C)	111.50±0.00	104.84±0.00	108.48±0.00
α_1 (K^{-1})	0.16±0.00	0.15±0.00	0.17±0.00
β_1 (K^{-2})	0.28±0.00	0.21±0.00	0.28±0.00
$\Delta C_{p,m,1}$ ($W.g^{-1}.K^{-1}$)	0.00±0.00	0.02+0.00	0.00±0.00
B ($W.g^{-1}$)	0.22±0.00	0.29±0.00	0.46±0.00
C ($mW.g^{-1}.K^{-1}$)	-4.45±0.03	-5.03±0.01	-2.79±0.02
D ($mW.g^{-1}.K^{-2}$)	0.01±0.00	-0.01±0.00	0.02±0.00
R^2	0.99	0.99	0.99

[a] The samples were heated at 5 ($K\ min^{-1}$) after calibration at the onset for the given weight and rate, in the case of single gels only one fitting peak is required (the error margins are from the nonlinear fitting), $\Delta C_{p,m}$ does not converge (Not available=NA) due to purely mathematical artefact, if the peaks are sufficiently apart with sufficient baseline tail on each side, the cumulative $\Delta C_{p,m}$ can be reliably determined via the $DSC_N(T)$ function for binary systems.

Table S11. Fit parameters and statistical coefficient of the $DSC_N(T)$ function fitted to the experimental DSC trace of 8mg of 5BA, 6BA, and $(5BA)_3(6BA)_1$ gels (20 wt%)[a].

**5BA6BA gels (20 wt%)**	**5BA gel**	**$(5BA)_3(6BA)_1$ gel**	**6BA gel**
ΔH_1 ($J.g^{-1}$)	Single peak	25.44±0.18	Single peak
$T_{m,1}^0$ (°C)		83.30±0.03	
α_1 (K^{-1})		0.04±0.00	
β_1 (K^{-2})		2.14±0.29	
$\Delta C_{p,m,1}$ ($W.g^{-1}.K^{-1}$)		NA	
ΔH_2 ($J.g^{-1}$)	36.12±0.01	119.88±0.49	53.04±0.01
$T_{m,2}^0$ (°C)	101.96±0.01	96.34±0.01	111.50±0.00
α_2 (K^{-1})	0.15±0.00	0.05±0.00	0.16±0.00
β_2 (K^{-2})	0.33±0.00	0.31±0.00	0.28±0.00
$\Delta C_{p,m,2}$ ($W.g^{-1}.K^{-1}$)	0.01±0.00	NA	0.00±0.00
B ($W.g^{-1}$)	0.54±0.00	0.23±0.01	0.22±0.00
C ($mW.g^{-1}.K^{-1}$)	-0.86±0.01	-2.64±0.03	-4.45±0.03
D ($mW.g^{-1}.K^{-2}$)	0.03±0.00	-0.01±0.00	0.01±0.00
R^2	0.99	0.99	0.99

[a] The samples were heated at 5 (K min^{-1}) after calibration at the onset for the given weight and rate, in the case of single gels only one fitting peak is required (the error margins are from the nonlinear fitting), $\Delta C_{p,m}$ does not converge (Not available=NA) due to purely mathematical artefact, if the peaks are sufficiently apart with sufficient baseline tail on each side, the cumulative $\Delta C_{p,m}$ can be reliably determined via the $DSC_N(T)$ function for binary systems.

Table S12. Fit parameters and statistical coefficient of the $DSC_N(T)$ function fitted to the experimental DSC trace of 8mg of 5BA, 10BA, and $(5BA)_3(10BA)_1$ gels (20 wt%)[a].

**5BA10BA gels (20 wt%)**	**5BA gel**	**$(5BA)_3(10BA)_1$ gel**	**10BA gel**
ΔH_1 ($J.g^{-1}$)	Single peak	39.84±0.31	Single peak
$T_{m,1}^0$ (°C)		83.91±0.03	
α_1 (K^{-1})		0.03±0.00	
β_1 (K^{-2})		1.46±0.17	
$\Delta C_{p,m,1}$ ($W.g^{-1}.K^{-1}$)		NA	
ΔH_2 ($J.g^{-1}$)	36.12±0.01	163.20±0.78	47.88±0.01
$T_{m,2}^0$ (°C)	101.96±0.01	97.39±0.01	108.48±0.00
α_2 (K^{-1})	0.15±0.00	0.05±0.00	0.17±0.00
β_2 (K^{-2})	0.33±0.00	0.27±0.00	0.28±0.00
$\Delta C_{p,m,2}$ ($W.g^{-1}.K^{-1}$)	0.01±0.00	NA	0.00±0.00
B ($W.g^{-1}$)	0.54±0.00	0.13±0.02	0.46±0.00
C ($mW.g^{-1}.K^{-1}$)	-0.86±0.01	1.81±0.05	-2.79±0.02
D ($mW.g^{-1}.K^{-2}$)	0.03±0.00	-0.01±0.00	0.02±0.00
R^2	0.99	0.99	0.99

[a] The samples were heated at 5 (K min^{-1}) after calibration at the onset for the given weight and rate, in the case of single gels only one fitting peak is required (the error margins are from the nonlinear fitting), $\Delta C_{p,m}$ does not converge (Not available=NA) due to purely mathematical artefact, if the peaks are sufficiently apart with sufficient baseline tail on each side, the cumulative $\Delta C_{p,m}$ can be reliably determined via the $DSC_N(T)$ function for binary systems.
